# Coupled-oscillator-humanizer revealed possible ensemble players’ ability to discriminate cross-correlation structures in auditory sequences of paired drum tapping

**DOI:** 10.1371/journal.pone.0336778

**Published:** 2025-11-20

**Authors:** Masahiro Okano, Sotaro Kondoh, Wataru Kurebayashi, Ryosuke O. Tachibana

**Affiliations:** 1 Graduate School of Human Development and Environment, Kobe University, Kobe, Hyogo, Japan; 2 Research Organization of Science and Technology, Ritsumeikan University, Kusatsu, Shiga, Japan; 3 Graduate School of Media and Governance, Keio University, Fujisawa, Kanagawa, Japan; 4 Faculty of Environment and Information Studies, Keio University, Fujisawa, Kanagawa, Japan; 5 Japan Society for the Promotion of Science, Chiyoda, Tokyo, Japan; 6 Graduate School of Information Science and Technology, The University of Osaka, Suita, Osaka, Japan; 7 Human Informatics and Interaction Research Institute, National Institute of Advanced Industrial Science and Technology (AIST), Tsukuba, Ibaraki, Japan; Universidad Nacional de Tres de Febrero, ARGENTINA

## Abstract

Natural fluctuations in the timing sequence are essential for a dynamic and expressive rendition of music. Various studies have demonstrated the statistical structure of such timing fluctuations in solo music performances and listeners’ ability to perceive them. However, the listening ability of timing structures in ensemble performances involving multiple players remains unclear despite its importance in understanding actual music skills. Here, we assessed listeners’ ability to distinguish the statistics of timing variations that resembled mutual adaptations between a pair of tapping players as a simplified example of ensemble performance. We created sound stimuli in which the drum-tapping timing sequences fluctuated either cooperatively among the pair or randomly using a generative model for synchronized pair tapping. Listing tests to discriminate between these stimuli, and a questionnaire on music experience and sophistication were conducted as a web-based experiment. Consequently, a trend was observed where participants with ensemble experience could discriminate between stimuli, whereas participants without ensemble experience could not. This discrimination ability did not correlate with the musical sophistication index. These results suggest that listeners with a certain music experience, particularly those involved in ensemble performances, can perceive the individuality of each player and the coordination between them based on the timing sequence structure.

## Introduction

In musical performances, mechanical precision similar to that of a metronome is not always desirable. This tendency is evident in Western classical music performances, particularly within the Romantic repertoire. The intentional manipulation of timing by a performer for expressive purposes is termed expressive timing, with prior research identifying characteristic strategies such as phrase-final lengthening [1–3] and phrase-arching [4,5]. Several studies have examined expressive timing in ensemble performances and revealed techniques such as primary voice leading and melody lead, as well as their effects on auditory impressions [1,6,7]. In contrast to Western classical music, the rock and pop genres are typically performed isochronously. Even in such genres, expressive or groovy performance requires micro-fluctuations in timing (micro-timing), i.e., slight deviations from the exact notated (or quantized) timing [8–11]. This timing fluctuation has attracted attention in performance research on various music genres [12–17]. Even in computer-based plays or drum machines, a certain amount of fluctuation is often added to the note timing to mimic human performance, and such programs are called “humanizers” [18]. The present study aims to extend the theoretical framework of the dynamical properties (time evolution structures of a system that cannot be captured simply by using the mean or variance of the fluctuation) of micro-timing in isochronous genres to ensemble situations by integrating it with the theory of coordination dynamics between partners, previously explored in paired tapping tasks. Furthermore, we investigated whether humans possess the perceptual ability to discriminate between coordination dynamics.

Intrinsic and natural fluctuations in musical performance have been widely observed in human cognition and behavior, and have been associated with the ‘$1/f^{\beta }$ ’ statistical structure [19–26]. The $1/f^{\beta }$ is a characteristic of the spectral pattern, which reflects the randomness and unpredictability of the fluctuations [24]. The *β* represents a linear slope of the log-log power spectrum, where *β* = 0 corresponds to white noise and *β* = 1 to pink noise [22,27]. Interestingly, listeners prefer music renditions with fluctuating structures closer to pink noise than to white noise [18,28]. This suggests that we can discriminate, to some extent, the $1/f^{\beta }$ structure that the auditory series contains.

Because the $1/f^{\beta }$ structure is an indicator that characterizes a single time series, it was applied to model the fluctuations in the solo performance. However, for the ensemble performance, the characteristics of the fluctuation structures remain unknown despite several related studies. For example, Hennig (2014) modeled ensemble performance as a mutually interacting complex system (MICS) and demonstrated that this model generated a naturally sounding “Billie Jean” performance [29]. This was achieved by simulating timing fluctuations through a synchronized tapping task in pairs, akin to a coupled oscillator with $1/f^{\beta }$ noise. Konvalinka et al. (2010) investigated rhythmic coordination in paired synchronized tapping tasks, finding that inter-tap interval (ITI) time series displayed a characteristic coordination dynamics in which players alternated between long and short intervals by referencing each other’s previous taps, resulting in positive lag ± 1 and negative lag 0 cross-correlation coefficients [30]. Okano et al. (2017) and Okano et al. (2019) replicated these previous findings across various tempos and observed a consistent pattern in ensemble coordination, even when tapping periods were extended [31,32]. These studies provide insights into the complex interplay between timing and coordination in ensemble performances and contribute to our understanding of how these dynamics are perceived and maintained. Okano et al. (2019) demonstrated that simulations of their model reproduced coordination dynamics [31]. Their model was formulated as a coupled oscillator model with a hybrid continuous-time/discrete-reset structure and had four parameters for each partner: phase and period correction parameters *a* and *b*, respectively; rebound strength *k* to an initial tempo; and timing noise *σ* (see Methods). They also demonstrated that their model replicates the multi-scale dynamics of the fluctuation and coordination of the partners’ ITIs and that these dynamics are modulated by a period-correction parameter (*b*). This appears to reflect the individuality of coordination among partners [31].

If the timing fluctuations and coordination of partners in an ensemble exhibit individuality and preference, as in the case of solos, humans should be able to discriminate between them. The perception of statistical structures in timing sequences may be affected by the ability to perceive rhythmic sounds and/or prior experience with such sound patterns. Recent studies on human rhythmic coordination suggest that humans can not only perceive and realize local beat synchronization but also adjust the global (dynamical) properties of variations, such as the β. of the $1/f^{\beta \;}\;$ structure, in behavioral rhythms to match external rhythms [33–41]. If, as discussed above, the dynamical properties of fluctuations are linked to the perception of performance, the ability to discern how these fluctuations are coordinated among partners would be crucial for an ensemble’s success.

What mechanisms are responsible for these perceptual abilities? One potential explanation for this is the accurate detection of timing fluctuations and asynchrony. However, previous research suggests that listeners do not perceive fluctuations or asynchrony with sufficient accuracy. The just noticeable difference for sound onset interval variations in isochronous sequences is reported to be approximately 6–10 ms for sequences with 240 ms intervals, and approximately 2.5 to 5% for sequences with lger intervals [42,43]. In addition, in the perception of asynchrony, the temporal order becomes imperceptible for time differences smaller than 15 to 35 ms [44,45]. These findings highlight the need to consider alternative mechanisms without presuming the precise detection of local fluctuations and asynchronies.

In this regard, the dynamical systems approach offers a compelling framework by treating an ensemble as a collective unit rather than as a sum of individuals. This approach suggests that ensemble coordination arises from anticipation and adaptation, grounded in self-other integration, and the collective behavior of the entire group, rather than relying on pairwise perceptual information processing [46–48]. Research adopting this approach has identified multiple factors influencing the coordination of performance, including the performer’s intrinsic rhythms [49], visual cues from bodily movements [50–52], cognitive chunking [53] and social relationships [54]. In other words, the dynamical characteristics of an ensemble are perceived through its overall impression, reflecting the collective behavior of members, rather than through a detailed analysis of timing from moment to moment.

This study performed an online listening experiment to examine whether listeners could distinguish between paired ITI series with varying parameter settings as generated by Okano et al. ’s (2019) coupled oscillator model. Our purpose was not to assess whether the generated stimuli sounded “human-like,” but rather to determine whether humans could differentiate coordination structures. Additionally, we explored the relationships among discrimination ability, musical sophistication, and experience in ensemble participation. Although the auditory stimuli presented in the experimental task–paired drum tapping at almost equal intervals–lack variations in pitch, timbre, or amplitude, they capture the core rhythmic element of timing differences and adjustments between performers that arise in a duet, the minimum unit of an ensemble. Timing is an extremely important element in acoustic communication, including music [42]; even a difference of a few tens of milliseconds can affect perceptual quality [13,16]. Thus, investigating the relationship between the auditory discriminability of coordination dynamics and listening impressions using this model serves as an essential early step towards a scientific understanding of the micro-timing of ensembles in the isochronous genre of music.

## Methods

### Overview

**Fig 1:**
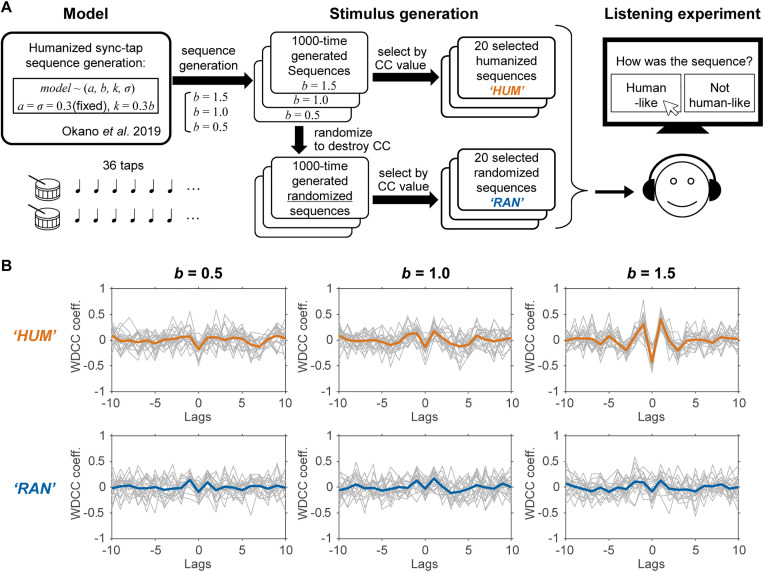
Overview of the methods and processes for generating experimental stimuli. (A) HUM stimuli were generated from the model with a consistent cross-correlation structure, and based on these HUM stimuli, RAN stimuli with a random cross-correlation structure were produced. These stimuli were sequentially presented to participants, who were asked to discriminate between them. (B) Correlation structures of the stimuli.

We conducted web-based listening experiments to assess the discrimination of the timing sequence stimuli of paired ITIs generated by the numerical simulation of synchronized tapping (**Fig 1**) using our previously reported model [31,55]. Three stimulus sequences were generated: humanized (HUM), randomized (RAN), and isochronous (ISO). Before starting the listening test, we presented HUM stimuli as examples of sounds containing “human-like” fluctuations, but RAN and ISO stimuli for showing “not human-like” fluctuation examples. After a brief practice session, the participants joined the test session and judged whether the presented stimulus was “human-like” or “not human-like.” Statistical analyses centered on the percentage of the responses that participants judged HUM and RAN stimuli to be “human-like” and musical experience.

The cross-correlation structure of the stimuli was examined using windowed detrended cross-correlation (WDCC) analysis [56]. While previous studies on solo performance and Hennig (2014) emphasized on the $1/f^{\beta }$ structure, the present study prioritized the cross-correlation structure because controlling stimulus properties by focusing on the $1/f^{\beta }$ structure should extend stimuli excessively. It is known that, in estimating $1/f^{\beta }$ structures, significant estimation errors arise unless there are at least 256 taps (data points) [27]. Several studies have suggested that WDCC is advantageous for investigating synchronization processes [31,34,56].

### Stimuli

Participants listened to the auditory stimuli using a web browser. The stimuli comprised mp3 audio files (S3 File), each containing 36 synchronized drum-tapping sounds produced by a pair of virtual players. In one experiment, participants were exposed to 20 HUM, 20 RAN, and 10 ISO stimuli throughout the example, practice, and test phases. All the stimuli were generated using MATLAB R2023a (MathWorks, USA). The stimuli were prepared according to the following procedure.

#### Humanized sequence (HUM).

Tap timing series were generated using the numerical simulation of the coupled oscillator model of Okano et al. (2019). In the model, the timing adjustment process in the paired synchronous tapping task was formulated as follows [31,57,55]:


$$\frac{d\theta_{i}\left(t\right)}{dt}=\omega_{i}\left(t\right)+\sigma_{i}\xi_{i}\left(t\right),$$ (1)

$$\frac{d\omega_{i}\left(t\right)}{dt}=0,\;\;\;\;\;\;\;\;\;\;\;\;\;\;\;\;\;\;\;\;\;\;\;\;\;\;\;$$ (2)

for i=1,2, where t is the time, $0\leq \theta_{i}\left(t\right)<2\pi$ and $\omega_{i}\left(t\right)>0$ are the phase and angular velocity of the i-th participant’s tapping, and $\xi_{i}\left(t\right)$ is the zero-mean Gaussian white noise of unit intensity, and $\sigma_{i}$ is the strength of the noise. When the i-th participant taps (i.e., $\theta_{i}\left(t\right)=2\pi$), the i-th participant’s phase $\theta_{i}$ is reset to 0, and the other (j-th) participant’s phase $\theta_{j}\left(t\right)$ and angular velocity $\omega_{j}\left(t\right)$ are reset as follows:


$$\theta_{j}\left(t\right)\leftarrow \theta_{j}\left(t\right)+a_{j}Z_{j}\left(\theta_{j}\left(t\right)\right),\;\;\;\;\;\;\;\;\;\;\;\;\;\;\;\;\;\;\;\;\;\;\;\;\;\;\;\;\;\;\;\;\;\;\;\;\;\;$$ (3)

$$\omega_{j}\left(t\right)\leftarrow \omega_{j}\left(t\right)+b_{j}Y_{j}\left(\theta \left(t\right)\right)-k_{j}\left(\omega_{j}\left(t\right)-\omega_{j}\left(0\right)\right),$$ (4)

where $Z_{j}\left(\theta_{j}\right)$ and $Y_{j}\left(\theta_{j}\right)$ are response functions to modulate the phase and the angular velocity, respectively, and $a_{j}$ and $b_{j}$ are gains for each function. The third term of eq (4) represents the intention to maintain the initial tempo, where $k_{j}$ is its gain.

We used the model parameters of *a* = 0.3, *k* = 0.3*b*, and *σ* = 0.3 for both players 1 and 2, with variations in *b* set at 1.5, 1.0, and 0.5 (these variations corresponded to the three participant recruitment announcements). These choices were made because the WDCC structure empirically depends on *b*: concretely, increasing *b* deepens the valley of lag-0 WDCC and raises the peak of lag ± 1 WDCC [31]. Based on this property, *b* = 1.5 was set as a relatively strong level of period correction, which generally reproduces the most prominent WDCC structures, as observed by Okano et al. (2019). The other two levels (*b* = 1.0 and 0.5) were set to represent moderate and weak periodic corrections, respectively (see Supplementary Table S1 in S1 Appendix A). *σ* and *k* modulated the variability of the ITI and asynchronies: these settings were set to produce a level of variability that would not allow a clear perception of the order of the two tap timings or the lengthening or shortening of the ITI (see Table 1). The resulting ITI series pairs were subjected to WDCC analysis to obtain the cross-correlation structure from lag −10 to +10. The generation of ITI series pairs was repeated 1000 times, and the 20 pairs with WDCC structures closest to the mean of the 1000 repetitions were selected as HUM stimuli and as the basis for RAN stimuli (Fig 1A).

**Table 1:** ITI and asynchrony for each stimulus group.

*b*	Stimuli	Player	Mean ITI	SD ITI	Mean asynchrony	SD asynchrony
0.5	Hum	1	494.28 ± 2.2	16.59 ± 1.22	0.07 ± 1.31	19.38 ± 2.55
		2	494.26 ± 2.06	16.54 ± 2.47		
	Ran	1	494.39 ± 2.2	18.11 ± 2.4	0.07 ± 1.31	19.38 ± 2.55
		2	494.15 ± 2.08	17.8 ± 2.54		
	Iso	1	499.84 ± 0.07	0.59 ± 0.05	0.00 ± 0.04	0.61 ± 0.04
		2	499.85 ± 0.07	0.58 ± 0.05		
1	Hum	1	493.93 ± 2.29	18.39 ± 2.48	−0.02 ± 0.9	18.36 ± 2.33
		2	493.97 ± 2.27	18.51 ± 2.42		
	Ran	1	493.95 ± 2.25	18.06 ± 2.63	−0.02 ± 0.9	18.36 ± 2.33
		2	493.95 ± 2.28	18.08 ± 2.47		
	Iso	1	499.78 ± 0.05	0.63 ± 0.07	−0.01 ± 0.04	0.69 ± 0.07
		2	499.77 ± 0.06	0.64 ± 0.07		
1.5	Hum	1	494.09 ± 1.76	19.95 ± 2.35	0.03 ± 1.07	20.15 ± 1.88
		2	494.21 ± 1.77	20.23 ± 1.71		
	Ran	1	494.24 ± 1.6	18.2 ± 2.18	0.03 ± 1.07	20.15 ± 1.88
		2	494.06 ± 1.93	18.27 ± 2.27		
	Iso	1	499.76 ± 0.09	0.65 ± 0.08	0.00 ± 0.04	0.76 ± 0.1
		2	499.77 ± 0.08	0.7 ± 0.08		

Note: The unit for all values except *b* is millisecond (ms). Mean ± SD is shown.

Note: The asynchronies of Player 2 are omitted. The means are the sign-reversed values of Player 1 and the SDs are the same.

#### Randomized sequence (RAN).

The RAN stimuli were generated from the HUM stimuli by shuffling the mean and standard deviation of the asynchronies between players in the HUM and RAN stimuli as follows:

(1) The *n*th asynchrony in the asynchrony series ***a*** = (*a*_1_, *a*_2, …_) was calculated using the following equation (hereafter, the bold type denotes a vector):

$$a_{n}=t_{n}^{\left(1\right)}-t_{n}^{\left(2\right)}$$

where $t_{n}^{\left(1\right)}$ and $t_{n}^{\left(2\right)}$ represent the *n*th tap timing series for Players 1 and 2, respectively.

(2) The average tap timing series ***m*** = (*m*_1_, *m*_2_, …) between the players was computed as follows:

$$m_{n}=0.5\times \left(t_{n}^{\left(1\right)}+t_{n}^{\left(2\right)}\right)$$

where *m*_*n*_ represents the *n*th average tap timing between players.

(3) The randomized tap timing series $t_{rand}^{\left(1\right)}$ and $\;t_{rand}^{\left(2\right)}$ for Players 1 and 2, respectively, were defined as follows:

$$t_{rand}^{\left(1\right)}=m+d_{rand}$$

$$t_{rand}^{\left(2\right)}=m-d_{rand}$$

where $d_{rand}$ represents a randomly sorted halved asynchrony series: for example, $d_{rand}=0.5\times \left(a_{11},\;a_{2},a_{6},\;\ldots \right)$.

(4) The first-order differences of $t_{rand}^{\left(1\right)},t_{rand}^{\left(2\right)}$ were calculated to obtain a randomized ITI series pair, which was input into the WDCC to derive the cross-correlation structure from lag –10 to lag + 10.
(5) Steps (3) and (4) were repeated 1000 times, and the 20 pairs of time-series $t_{rand}^{\left(1\right)}$ and $t_{rand}^{\left(2\right)}$ whose cross-correlation structure was closest to the average of the 1000 iterations were selected as the RAN stimuli. The cross-correlation structures of HUM and RAN used in the experiments are illustrated in **Fig 1B**. The WDCC coefficients for lag –1 to +1 in the resulting RAN stimuli are presented in Table S2 in S1 Appendix A.

#### Isochronous sequence (ISO).

The ISO stimuli were designed to be easily distinguishable from HUM and RAN, with their ITI fluctuations maintained below the perceptual threshold, and the partner’s taps were perfectly synchronized. This stimulus was used to identify participants who were not paying attention during the listening test. The generation method was similar to that of the RAN, except that the model parameter *σ* was set to *σ* = 0.01. The descriptive statistics for the mean and standard deviation of the ITI and asynchrony for HUM, RAN, and ISO are presented in **Table 1**. Note that the mean ITI of ISO was slightly longer than those of HUM and RAN because the degree of joint rushing decreased when the other parameters were fixed and sigma was reduced. This discrepancy between the ISO and the other stimuli was not problematic for our analysis because we designed this stimulus to detect inattentive participants.

#### Sound generation.

First, a template MIDI file was created using manual entries. The template comprised 36 pairs of snare taps at equal intervals (interbeat interval = 500 ms) by two players in perfect synchronization. The timing of these taps was replaced by the tap timing series generated using the aforementioned procedure with a MATLAB MIDI toolbox [58]. The MIDI files were subsequently converted into WAV files using FluidSynth (https://www.fluidsynth.org/), part by part. The output WAV files were combined and re-exported as a single stereo WAV file using MATLAB. Different sound fonts were used for Player 1 (FluidR3_GM, https://github.com/urish/cinto/tree/master) and Player 2 (GeneralUserGS, https://schristiancollins.com/generaluser.php) to prevent unnatural sound localization. Additionally, the left channel of the WAV file contained waveforms synthesized from Players 1 and 2 with an amplitude ratio of 4:1, whereas the right channel contained waveforms synthesized with the opposite amplitude ratio.

### Participant recruitment

The participants in the online listening experiments were recruited through a crowdsourcing platform (CrowdWorks, Inc.). They applied to three recruitment announcements posted on the platform and participated in one or more of the experiments. Each announcement corresponded to a distinct experimental condition defined by varying the model parameters of the stimuli. The recruitment period for all announcements was from 05/03/2024–10/03/2024. For each recruitment announcement, 150 participants participated in the experiment, with some participating in more than one experiment. Participants who participated in multiple experiments were identified, and all the participants were assigned unique IDs.

This study was approved by the Ethics Committee of the Graduate School of Human Development and Environment of Kobe University (approval number: 697). The participants were provided a detailed explanation of the research and written informed consent was obtained from all the participants, who clicked on a checkbox to approve before starting the tasks on the website for the experiment.

### Music sophistication questionnaire

We used a musical sophistication questionnaire, the Goldsmith Musical Sophistication Index (G-MSI), in the Japanese language [59,60] to assess the relationship between participants’ abilities for music perception or the performance and perception of the statistical structure of timing sequences. This index is a widely used measure that has been translated into various languages since its release in 2014; it has demonstrated good psychometric properties and correlation with performance on listening tests that measure two different abilities: melodic memory and beat perception [59,60]. We calculated these scores from the responses according to a previous report [60]. However, methods for measuring perceptual abilities related to the ensemble are currently limited to beat alignment tests [61–63], that is, tests related to the judgment and realization of synchrony between beats (or beats and actions). Thus, in the present study, we also asked participants about their experiences of habitual participation in ensembles (yes/no selection and, if yes, genre selection) for an exploratory analysis.

### Experimental procedure

Participants registered for the experiment on the crowdsourcing platform and received a URL directing them to the experiment website created using lab.js [64]. Initially, the screen displayed the experiment description and a checkbox for providing informed consent. Upon agreeing and proceeding, the participants were asked questions regarding their age, gender, and audio playback environment.

Next, the participants adjusted the sound volume according to the on-screen instructions. They first set the volume to 25% on their computer and adjusted it to a comfortable level while listening to the same drum-tapping sounds used in the experiment. Subsequently, a headphone screening test was conducted to confirm whether the participants used headphones or earphones [65]. The participants listened to three tones and selected the weakest one. The three tones were 200-Hz pure tones but with (i) diotic, (ii) diotic and 6-dB softer, and (iii) dichotic antiphase presentations. This test was repeated six times, and only participants who answered correctly at least five times were allowed to proceed to the subsequent stages.

An explanation of the experimental task was then provided, which included an example of an auditory stimulus and the following instructions: “The sound you just heard is a computer-generated replica of two players playing a snare drum together.” “In the experiment, you will be asked to judge whether the fluctuations in the timing of the drum sound resemble those of a human playing or not.” Following these instructions, HUM stimuli were presented as an example of “human-like” fluctuations, while RAN and ISO stimuli were presented as examples of “not human-like” fluctuations, twice, twice, and once, respectively.

Subsequently, the participants completed the practice trials. During the practice trials, the participants were instructed to listen to a stimulus and click the “human-like” button if they perceived it as HUM, and “not human-like” button if they perceived it as RAN or ISO within 2.5 s. The practice comprised nine trials, with HUM, RAN, and ISO presented three times each in a random order. The five HUM–RAN pairs used in the examples and practice were selected to represent the range of the cross-correlation structures of the HUM presented in the main task. These stimuli corresponded to the 1^st^, 5^th^, 10^th^, 15^th^, and 20^th^ ranks when the 20 stimuli were sorted by their distance from the average cross-correlation structure (calculated as the sum of the squared difference from the ensemble mean of the WDCC function) output from the model simulations. The participants were instructed to keep their eyes on a gaze point at the center of the monitor while listening to the stimuli. After the button response, they received feedback on whether their answers were correct or incorrect during the practice trials. Regardless of the correct response rate in the practice trials, the participants proceeded to the main experiment without being screened after completing one set of practice trials. This was because the extent to which HUM and RAN could be discriminated at the beginning was unclear.

The main experiment comprising three blocks of 12 trials was then conducted. In each block, five HUM and RAN stimulus trials and two ISO stimulus trials were presented in a random order. The participants were asked to judge whether the sound they heard was HUM or otherwise (if the former, they selected “human-like;” if the latter, they selected “not human-like”) within 2.5 s. We employed the two-alternative forced-choice paradigm to detect subtle differences in listeners’ perceptions and obtain perceptual bias and sensitivity according to signal detection theory. The participants were instructed to keep their eyes on a gaze point at the center of the monitor while listening to the stimuli. They were allowed to take breaks of any length between the blocks. After completing all trials, the participants were asked to respond to all the questions in the Japanese version of the G-MSI [59,60]. They were also asked to specify whether they had ever engaged in regular group musical activities, the genre of music they had experienced, and the duration of their involvement in years. The web system provided a completion code to each participant after they completed these questionnaires.

### Data screening

In online experiments, participants can participate at any location and time, making it impossible for the experimenter to monitor their behavior. This raises concerns about dishonest or careless response behaviors (satisficing) [66,67]. To ensure that the analyses were performed only for participants who followed the instructions, data were excluded if any of the following criteria were met: the participant reported recognizing noise sources other than their computer, provided an answer other than the name or model number of headphones or earphones in the audio playback environment question, ran out of time in the main task twice or more, or judged ISO as “human-like” twice or more (The ISO is easily discriminable, and it is only presented six times during the test phase. In fact, the number of errors of judging the ISO as “human-like” was once or less for more than 90% of the participants: see Table S3 in S1 Appendix A). The numbers of participants who passed the screening were 100, 100, and 87 for *b* = 1.5, 1.0, and 0.5, respectively. The demographics are presented in **Table 2** (see Table S4 in S1 Appendix A for the music genres of the participants who passed the screening). Participants who participated in multiple *b* conditions were identified using their user ID on the crowdsourcing platform. The number of unique participants who passed the screening was 181:34 participants participated in all conditions; 53 participants participated in *b* = 1.5 and *b* = 1.0 conditions; 47 participants participated in *b* = 1.5 and *b* = 0.5 conditions; 40 participants participated in *b* = 1.0 and *b* = 0.5 conditions; 34, 41, and 34 participants participated in only *b* = 1.5, *b* = 1.0, and *b* = 0.5 conditions, respectively.

**Table 2:** Demographics of participants who passed the screening.

*b*		1.5				1.0				0.5			
Sex		male		female		male		female		male		female	
Ensemble		Yes	No	Yes	No	Yes	No	Yes	No	Yes	No	Yes	No
N		7	44	14	34	10	46	14	30	9	38	13	27
Age (year)	M	37.3	38.1	43	38.1	36.1	39.2	39.4	38.2	46.9	43.4	36.5	36.5
	SD	12.4	9.0	9.4	6.9	11.8	7.8	7.2	8.9	10.8	8.8	7.9	9.7
G-MSI — All	M	171	117	132	120	163	121	146	115	152	119	141	126
	SD	26.2	31.1	32.1	35.8	21.5	30.5	32.3	35.7	25.4	30.7	40.1	30.7
Active	M	33.9	26.1	27.6	25.7	31.6	26.6	28.6	24.9	29.9	27.4	30.5	26.6
	SD	7.0	8.0	10.5	7.5	7.4	7.9	10.7	8.6	6.0	7.7	10.3	7.7
Perception	M	49.1	33.6	35.4	32.7	47.3	34.1	40.4	31.1	43.4	32.7	36.3	34
	SD	10.3	10.5	7.9	11.1	8.3	9.0	8.3	8.8	10.9	9.8	9.6	9.4
Training	M	23.6	11.7	22.1	15	23.6	13	23.1	14	20.7	11.3	22.6	14.8
	SD	8.0	5.1	7.4	8.0	5.5	6.6	7.4	8.3	7.3	5.3	8.0	7.0
Singing	M	31.1	19.5	19.5	19.4	29.5	20.1	25.3	18.1	26.6	18.8	21.8	20.8
	SD	10.1	8.1	7.8	8.8	7.0	8.5	7.9	9.2	8.3	8.1	10.3	6.8
Emotion	M	32.3	25.3	25.9	26.5	31.1	25.9	28.1	25.4	30.4	26.8	27.5	28.3
	SD	3.1	6.4	8.1	6.3	4.5	6.0	7.8	7.6	4.0	6.3	8.3	6.8

Note: Additionally, there was one person with no gender response or ensemble experience in *b* = 1.5.

### Statistical analyses

The analyses were conducted in an exploratory manner, focusing on whether the proportion of “human-like” responses, along with sensitivity and bias of discrimination (*d*’ and *C*, respectively, in signal detection theory [68]), varied depending on the stimulus, parameter *b*, and participant profile. The *d*’ and *C* are derived from the proportion of “human-like” responses to HUM and RAN. A larger positive *d*’ indicates a higher percentage of “human-like” responses to HUM than to RAN, whereas a larger negative *d*’ indicates the opposite. A larger positive *C* suggests a bias towards “human-like” responses, whereas a larger negative *C* indicates the reverse. The *d*’ and C were calculated using MATLAB R2023a (Mathworks, USA). The S4 Dataset contains data used for statistical analyses, including data removed during screening.

We adopted a linear mixed model (LMM: sum contrast coding) in all the statistical analyses because fixed effects in the LMM have been empirically demonstrated to be robust to violations such as homogeneity of variance, normality of residuals, and failure to estimate random effects [69,70]. In our data, all random effects could not be estimated and Levene’s test and Kolmogorov–Smirnov test suggested violations of the homogeneity of variance and normality of residuals in some parts of the analyses (see Results). Thus, we adopted LMM analyses and, for reference, showed the results using robust standard errors (Table S5-S9 in S1 Appendix A). LMM analyses were performed using the lme4 [71] and lmerTest packages [72] in R4.2.2 (R Core Team). All the LMM models were fitted with a restricted maximum likelihood (REML), and significance was calculated using Satterthwaite’s method for estimating the degrees of freedom and *p*-values. The significance level was set at *p* < .05. When analysis of variance (ANOVA) detected effects above borderline significance (*p* < .10), *post hoc* power analyses were performed using the simr package [73], and a 95% confidence interval was reported (number of simulations: 1000). Additionally, the effect sizes (standardized fixed effect coefficients and inclusive R^2^ [74]) and robust standard errors for fixed effect estimates were calculated using the effectsize [75], partR2 [73] (number of bootstraps: 1000) and clubSandwich [76] packages.

As demonstrated in S2 Appendix B, the correlations between the G-MSI score and the accurate response rate in practice trials, *d*’, or *C* were weak and inconsistent. Meanwhile, participants with ensemble experience generally exhibited a higher total G-MSI scores than those without such experience (Table 2). A linear mixed model analysis was performed to determine whether the total G-MSI scores differed across ensemble experiences (yes and no) and levels of parameter *b* (1.5, 1.0, and 0.5). The lme4 model formula was G-MSI score ~ *b* * ensemble experience + (1 | participant ID). As discussed in the **Results** section, the fixed effect of ensemble experience was significant, prompting subsequent analyses focused on the influence of ensemble experience.

We compared the proportion of “human-like” responses to HUM and RAN (the number of “human-like” responses divided by the number of responses that did not run out of time) using LMM to assess the extent to which HUM and RAN were discriminated. The fixed-effect variables included stimulus type (HUM and RAN), parameter *b* (0.5, 1.0, and 1.5), and ensemble experience (yes or no). Participant ID was included as a random effect variable. The lme4 model formula was the proportion of “human-like” response ~ stimulus type * *b* * ensemble experience + (1 | participant ID). ISO was excluded from the analysis because it was specifically designed to be easily distinguished to identify satisficing and inattentive participants. Furthermore, *d*’ and *C* were analyzed using LMM, with *b* and ensemble experience as fixed effect variables and participant ID as a random effect variable. The lme4 model formulas were *d*’ ~ *b* * ensemble experience + (1 | participant ID) and *C* ~ *b* * ensemble experience + (1 | participant ID). In addition, to examine the effects of ensemble experience on the practice trials, the accurate response rate in the practice trials was analyzed using LMM, with parameter *b* and ensemble experience as fixed effect variables and participant ID as a random effect variable. The lme4 model formula was accurate response rate ~ *b* * ensemble experience + (1 | participant ID).

## Results

### Ensemble experience and G-MSI scores

To examine whether the total G-MSI score varied across experimental conditions or ensemble experience, an LMM analysis was conducted with the total G-MSI score as the dependent variable, and *b* and ensemble experience as fixed effects variables. The model converged (REML criterion = 2418.5; marginal R^2^ [95% CI] =.176 [0.082, 0.258]). The Levene’s test did not suggest a significant violation of the assumption of homogeneity of variances: *F*(5, 281) = 0.497, *p* = .778). The Kolmogorov–Smirnov test suggested a significant violation of the normality of the residuals: *D* = 0.155, *p* < .001. The ANOVA revealed a significant effect of ensemble experience (*F*(1.00, 180.24) = 39.42, *p* < .001, $\eta_{p}^{2}$ = .179, 95% CI of simulated power = 99.63–100.0%), suggesting that participants with ensemble experience had significantly higher total G-MSI scores than those without it. The main effect of *b* and interaction between *b* and ensemble experience were not significant (*F*(2.00, 107.42) = 2.15, *p* = .121, $\eta_{p}^{2}$ = .039; and *F*(2.00, 107.42) = 0.14, *p* < .866, $\eta_{p}^{2}$ = .003, respectively).

The fixed effect estimates are presented in Table 3 (standardized and robust estimates and inclusive R^2^ are presented in Table S5 in S1 Appendix A). In addition to the effects of the ensemble experience, participants in the *b* = 0.5 condition exhibited significantly higher total G-MSI scores than those in the other conditions (*t*(107.51) = 2.07, *p* = .040). The other fixed effects are not significant (*ps* > .05).

**Table 3:** Fixed effect estimates on total G-MSI score.

Term	Estimate	SE	*df*	*t*	*p*
Intercept	136.63	2.58	180.24	52.88	<.001^*^
*b* (0.5)	1.33	0.64	107.51	2.07	.040^*^
*b* (1.0)	–0.77	0.65	108.17	–1.19	.238
Ensemble (no)	–16.22	2.58	180.24	–6.28	<.001^*^
*b* (0.5) × Ensemble (no)	–0.32	0.64	107.51	–0.50	.615
*b* (1.0) × Ensemble (no)	0.08	0.65	108.17	0.12	.903

* : *p* < .05.

### Responses to the HUM and RAN stimuli

**Fig 2:**
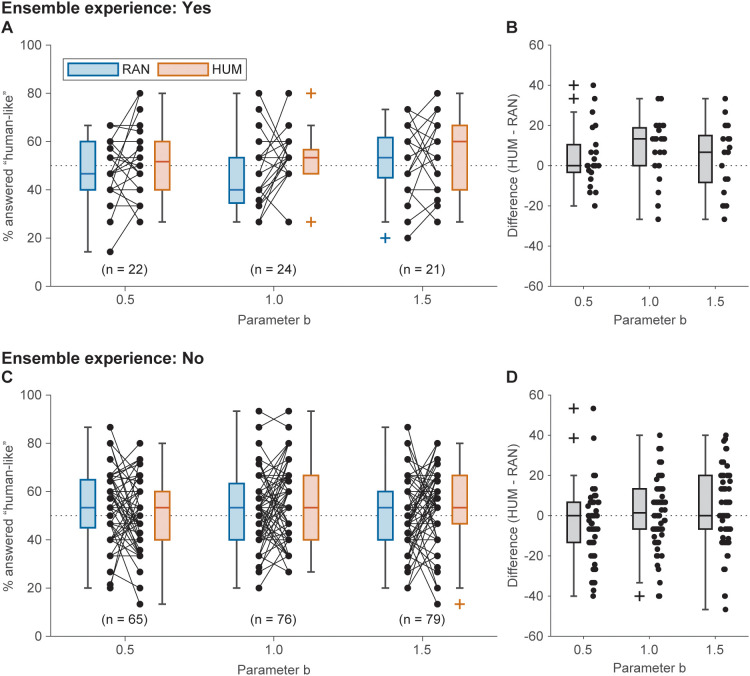
Comparison of the percentage of “human-like” responses by group and stimulus. chance levels Panels **A** and **B** display the data for participants with ensemble experience, whereas panels **C** and **D** show the data for participants without ensemble experience. (**A**) and (**C**): box charts illustrating the percentages of participants’ responses as “human-like” to the RAN (blue) and HUM (red) stimuli, with adjacent dots representing individual participant data. (**B**) and (**D**): box chart for the difference between the percentage of “human-like” responses to RAN and HUM, with neighboring dots indicating each participant’s data. The dashed lines indicate chance levels (50% for panels **A** and **B**, and 0 for **C** and **D**).

Fig 2 presents the percentage of “human-like” responses for HUM and RAN, alongside the chance level (50%, as participants chose between “human-like” and “not human-like” in all trials). To examine whether HUM obtained more “human-like” responses than RAN, we performed a LMM analysis with the “human-like” response rate as the dependent variable and stimulus type, *b*, and ensemble experience as independent variables. The model converged (REML criterion: 4609.9). The Levene’s test did not suggest a significant violation of the assumption of homogeneity of variances: *F*(11, 562) = 1.60, *p* = .094. The Kolmogorov–Smirnov test did not suggest a significant violation of the normality of the residuals: *D* = 0.02, *p* = .913. The ANOVA revealed a significant effect of stimulus type (*F*(1.00, 384.73) = 5.50, *p* = .020, $\eta_{p}^{2}$ = .014, 95% CI of simulated power = 38.92–45.13%) and borderline interaction of stimulus type and ensemble experience (*F*(1.00, 384.73) = 3.79, *p* = .052, $\eta_{p}^{2}$ = .010, 95% CI of simulated power = 47.15–53.44%). Other main effects and interactions were not significant (*b*: *F*(2.00, 554.60) = 0.74, *p* = .476, $\eta_{p}^{2}$ = .003; ensemble experience: *F*(1.00, 172.17) = 1.88, *p* = .172, $\eta_{p}^{2}$ = .011; stimulus type × *b*: *F*(1.00, 384.73) = 3.79, *p* = .052, $\eta_{p}^{2}$ = .010; *b* × ensemble experience: *F*(2.00, 554.60) = 0.38, *p* = .683, $\eta_{p}^{2}$ = .001; stimulus type × *b* × ensemble experience: *F*(2.00, 384.73) = 1.07, *p* = .344, $\eta_{p}^{2}$ = .006). These findings suggest that participants with ensemble experience were more likely to perceive HUM as “human-like” than RAN compared to participants without ensemble experience.

The fixed effects estimates are shown in Table 4 (standardized and robust estimates and inclusive R^2^ are presented in Table S6 in S1 Appendix A). HUM obtained significantly more “human-like” responses overall (*t*(384.73) = 2.35, *p* = .020). Furthermore, a borderline interaction between stimulus type and ensemble experience was revealed: participants without ensemble experience provided fewer “human-like” responses to HUM (*t*(384.73) = –1.95, *p* = .052). The other fixed effects were not significant (*ps* > .10).

**Table 4:** Fixed effect estimates on “human-like” response rate.

Term	Estimate	SE	*df*	*t*	*p*
Intercept	52.26	0.97	172.17	53.76	<.001^*^
Stimuli (HUM)	1.37	0.59	384.73	2.35	.020^*^
*b* (0.5)	–0.30	0.97	555.37	–0.31	.758
*b* (1.0)	–0.82	0.96	560.77	–0.85	.394
Ensemble (no)	1.33	0.97	172.17	1.37	.172
Stimuli (HUM) × *b* (0.5)	–1.23	0.84	384.73	–1.47	.143
Stimuli (HUM) × *b* (1.0)	0.93	0.81	384.73	1.14	.256
Stimuli (HUM) × Ensemble (no)	–1.14	0.59	384.73	–1.95	.052^†^
*b* (0.5) × Ensemble (no)	–0.18	0.97	555.37	–0.18	.853
*b* (1.0) × Ensemble (no)	0.79	0.96	560.77	0.83	.408
Stimuli (HUM)× *b* (0.5) × Ensemble (no)	–0.91	0.84	384.73	–1.09	.278
Stimuli (HUM) × *b* (1.0) × Ensemble (no)	–0.26	0.81	384.73	–0.32	.749

^†^ : *p* < .10; * : *p* < .05.

### Ensemble experience, sensitivity, and response bias

**Fig 3:**
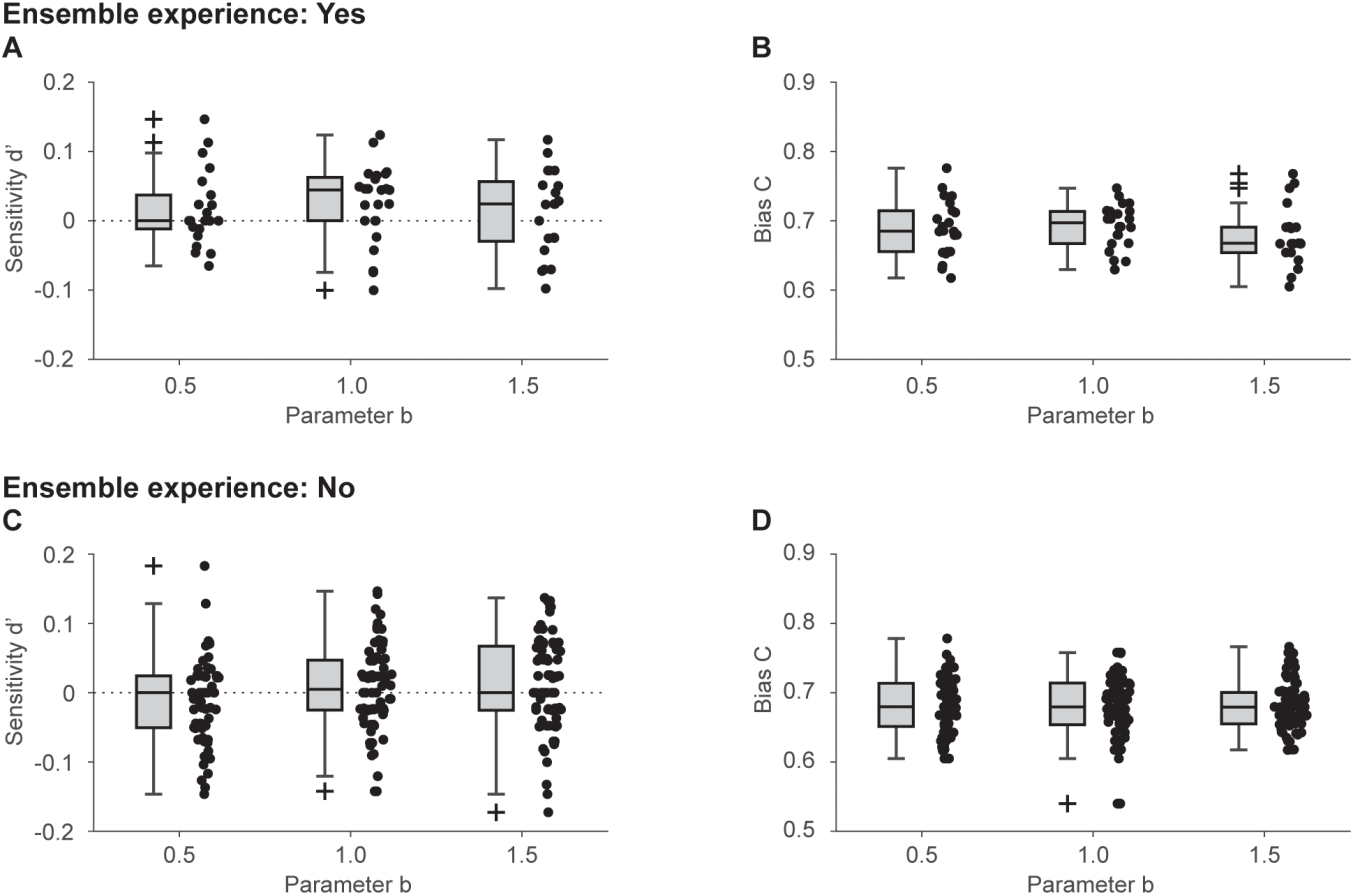
Comparison of sensitivity and response bias. Panels A and B present the data for participants with ensemble experience, whereas panels C and D present the data for those without ensemble experience. (A) and (C): box charts for sensitivity *d*’, with adjacent dots representing individual participant data. (B) and (D): box charts for bias *C*, with neighboring dots indicating each participant’s data.

To assess whether the group differences in the response tendencies observed in the above analysis stemmed from sensitivity or response bias, LMM analyses were conducted on *d*’ and *C* as dependent variables, with *b* and ensemble experience as fixed effects variables. Fig 3 illustrates the distributions of *d*’ and *C*. Both models converged (REML criterion: −740.1 for *d*’ and −1002.1 for *C*). The Levene’s test did not suggest a significant violation of the assumption of homogeneity of variances: *F*(5, 281) = 0.74, *p* = .591 for *d*’, and *F*(5, 281) = 1.44, *p* = .201 for *C.* The Kolmogorov–Smirnov test did not suggest a significant violation of the normality of residuals: *D* = 0.05, *p* = .445 for *d*’ and *D* = 0.06, *p* = .219 for *C*. The ANOVA on *d*’ revealed the borderline main effect of ensemble experience (*F*(1.00,281.00) = 3.39, *p* = .066, $\eta_{p}^{2}$ = .012, 95% CI of simulated power = 40.30–46.54%). The main effect of *b* and interaction between *b* and ensemble experience were not significant (*b*: *F*(2.00,281.00) = 1.03, *p* = .357, $\eta_{p}^{2}$ = .007; *b* × ensemble experience: *F*(2.00,281.00) = 1.00, *p* = .371, $\eta_{p}^{2}$ = .007). Conversely, the ANOVA on *C* revealed that all the main effects and interactions were not significant (*b*: *F*(2.00, 162.90) = 0.93, *p* = .396, $\eta_{p}^{2}$ = .011; ensemble experience: *F*(1.00, 171.75) = 2.05, *p* = .154, $\eta_{p}^{2}$ = .012; *b* × ensemble experience: *F*(2.00, 162.90) = 0.38, *p* = .687, $\eta_{p}^{2}$ = .005). Thus, although the evidence remains limited, group differences in response tendencies were more likely attributable to sensitivity rather than to response bias.

The fixed effect estimates for *d*’ are provided in Table 5 (standardized and robust estimates and inclusive R^2^ are presented in Table S7 in S1 Appendix A). Participants without ensemble experience demonstrated a slightly lower sensitivity than those with ensemble experience (*t*(281) = –1.84, *p* = .066). The fixed effect estimates for *C* are shown in Table 6 (standardized and robust estimates and inclusive R^2^ are presented in Table S8 in S1 Appendix A). No significant effects were observed across any of the terms (*ps* > .05).

**Table 5:** Fixed effect estimates of sensitivity *d*’.

Term	Estimate	SE	*df*	*t*	*p*
Intercept	0.01	0.00	281.00	2.24	.026^*^
*b* (0.5)	–0.01	0.01	281.00	–1.38	.168
*b* (1.0)	0.01	0.01	281.00	1.02	.309
Ensemble (no)	–0.01	0.00	281.00	–1.84	.066^†^
*b* (0.5) × Ensemble (no)	–0.01	0.01	281.00	–1.09	.275
*b* (1.0) × Ensemble (no)	0.00	0.01	281.00	–0.24	.808

^†^ : *p* < .10; * : *p* < .05.

**Table 6:** Fixed effect estimates on bias C.

Term	Estimate	SE	*df*	*t*	*p*
Intercept	0.68	0.00	171.75	196.58	.000^*^
*b* (0.5)	0.00	0.00	163.86	0.29	.772
*b* (1.0)	0.00	0.00	173.39	0.99	.325
Ensemble (no)	0.00	0.00	171.75	–1.43	.154
*b* (0.5) × Ensemble (no)	0.00	0.00	163.86	0.23	.821
*b* (1.0) × Ensemble (no)	0.00	0.00	173.39	–0.83	.406

* : *p* < .05.

### Accurate response rate in practice trials

**Fig 4:**
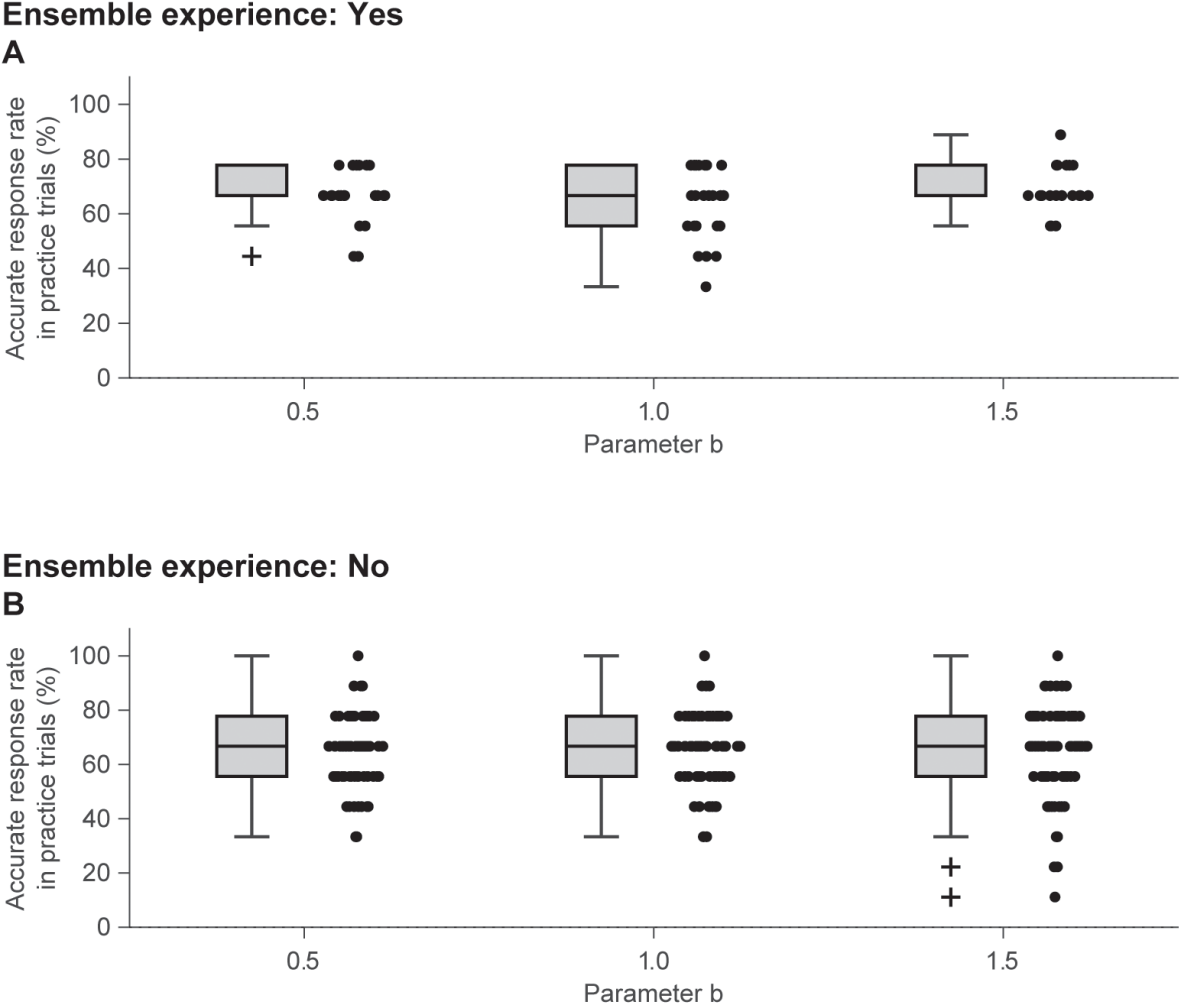
Comparison of accurate response rate in practice trials. Panels A and B present data for participants with and without ensemble experience, respectively, with adjacent dots representing individual participant data.

The effects of *b* and ensemble experience on the accurate response rate in the practice trials were analyzed using an LMM to check whether the responses of the participants with ensemble experience were accurate in the practice trials. The model converged (REML criterion: −275.80). The Levene’s test suggested a significant violation of the assumption of homogeneity of variances: *F*(5, 281) = 2.61, *p* = .025. The Kolmogorov–Smirnov test suggested a significant violation of the normality of the residuals: *D* = 0.12, *p* < .001. The ANOVA revealed that all the main effects and interactions were not significant (*b*: F(2.00, 281.00) = 1.16, *p* = .316, $\eta_{p}^{2}$ = .008; ensemble experience: *F*(1.00, 281.00) = 0.85, *p* = .357, $\eta_{p}^{2}$ = .003; *b* × ensemble experience: *F*(2.00, 281.00) = 0.96, *p* = .386, $\eta_{p}^{2}$ = .007), suggesting that participants with ensemble experience were not necessarily able to discriminate better from the practice stage, while the minimum accurate response rate appeared to be slightly high (Fig 4).

The fixed effect estimates are provided in Table 7 (standardized and robust estimates and inclusive R^2^ are presented in Table S9 in S1 Appendix A). None of the fixed effects was significant (*ps* > .05).

**Table 7:** Fixed effect estimates of the accurate response rate in practice trials.

Term	Estimate	SE	*df*	*t*	*p*
Intercept	0.66	0.01	281.00	66.90	<.001
*b* (0.5)	0.00	0.01	281.00	–0.02	.983
*b* (1.0)	–0.02	0.01	281.00	–1.32	.189
Ensemble(no)	–0.01	0.01	281.00	–0.92	.357
*b* (0.5) × Ensemble (no)	0.00	0.01	281.00	–0.15	.883
*b* (1.0) × Ensemble (no)	0.02	0.01	281.00	1.27	.205

## Discussion

This study aimed to examine whether it is possible to discriminate between paired ITI series generated by the model of Okano et al. (2019) (HUM) and paired randomized ITI series (RAN) and whether there is a relationship between discrimination ability and musical background. Overall, HUM elicited more “human-like” responses than RAN. In addition, although the statistical effect was borderline and the statistical power was limited, the results suggested that participants without ensemble experience had difficulty discriminating, whereas several participants with ensemble experience could discriminate to some extent. In addition, musical sophistication did not correlate with sensitivity or judgment bias.

The observed perceptual difference between HUM and RAN would reflect the difference in their cross-correlation structures. Previous studies on micro-timing have demonstrated that dynamical properties of timing can be distinguished despite the asynchrony and ITI differences contained in micro-timing being close to the perceptual threshold. The difference in the responses to HUM and RAN in the present study extends the scope of the findings of the previous studies to the level of coordination structures between partners. HUM were stimuli with mutual timing adaptation structures, whereas RAN were stimuli without consistent cross-correlation structures. Although the evidence is limited, the fact that these can be distinguished to a certain degree is a necessary requirement for the question of whether there is individuality and preference in the fluctuation of the timing and coordination of partners in an ensemble.

Differences in *b* in the HUM are linked to the strength of the mutual timing adaptation [30,31,57,55]; the larger *b*, the more the mutual timing adaptation pattern is strengthened. This initially led us to hypothesize that a larger *b* would make discriminating between HUM and RAN easier. However, the relationship between the magnitude of *b* and the rates of judgment, sensitivity, and bias was unclear. While a relatively small dependence between partners was sufficient for participants with ensemble experience to discriminate, a greater dependence did not necessarily facilitate discrimination within the scope of this study. Rather, although the group differences in sensitivity *d*’ were not statistically significant, they tended to be more pronounced in conditions with smaller *b*. This may be because RAN was judged to be more “human-like” on average than HUM, with *b* = 0.5, although this difference was not statistically significant in the group without ensemble experience. For these participants, *b* = 0.5 may be too small a dependence for judgment. The experimental design, in which *b* was an inter-subject factor, weakened the statistical power and made it difficult to draw definitive conclusions. Setting *b* as an intra-subject factor and more pronounced *b* values will contribute to validating these points.

This study suggests a possible connection between ensemble experience and the ability to discriminate between fluctuations and coordination in multipart auditory sequences. If so, participants with ensemble experience may have learned the difference between HUM and RAN through several practice sessions with feedback, or may have already had the potential for this. Although the participants with ensemble experience did not exhibit a significantly higher rate of accurate responses in the practice trials, they demonstrated a slightly higher minimum accurate response rate during the practice trials and slightly greater sensitivity during the main experiment. These results motivate further validation studies with a more precise grouping of ensemble experiences.

From a perceptual information processing perspective, our model captures mutual timing adaptation, which is a part of the cognitive-motor skills involved in ensemble performance. The cross-correlation structure of HUM stimuli is the outcome of a player’s mutual adaptation [48]. From this perspective, an ensemble experience may cultivate an understanding of the fluctuations driven by mutual timing adaptations among individual performers. Conversely, within the dynamical systems framework, ensemble coordination is interpreted as an emergent collective behavior arising from multiple coupled oscillators (performers) rather than resulting from short-term, pairwise interactions between individuals [76]. From this perspective, ensemble experience may nurture an understanding of fluctuations as a group-level collective behavior of performers, rather than individual adaptations.

However, it should be noted that mutual timing adaptation does not necessarily equate to high-quality coordination. For instance, Repp (2010) found that, in a sensorimotor synchronization experiment, participants with extensive musical experience showed slower phase correction responses, suggesting that faster phase correction is not always better for performance [77]. Further research is needed to explore the specific mechanisms or perspectives that enable discrimination between structured and random fluctuations, and how ensemble experience contributes to this ability. Furthermore, the “human-like” or “not human-like” labels used for judgment in this study were only operational. Therefore, future research should explore task instructions that more directly reflect the concept of “coordination.”

Scientific research on micro-timing in isochronous music ensembles remains in its early stages, necessitating investigations in minimal settings. Thus, this study focused on the duo, the smallest functional unit of ensembles, to examine the effects of the structures of timing fluctuation and coordination. Although the discriminability levels obtained in this study were at the borderline of statistical significance, further verification is needed. The results offer valuable foundational data for future research exploring additional factors. A future direction for ensemble micro-timing research is to extend the investigation to non-temporal dimensions. For example, a study demonstrated that a misalignment in the micro-timing of snare drums is rated worse than a similar misalignment in bass drums [77]. Human responses to timing errors are stronger for high-frequency tones than low-frequency tones [78]. Timbre affects perceptual note attacks and their onset [79]. How cognitive and biomechanical constraints, visual cues, and social interactions modulate the coordination structure of fluctuations remains unclear. Therefore, research using stimuli with higher ecological validity that considers these factors is necessary. In such cases, using a model based on the Kuramoto model may be effective [49,80]. These formulate temporal adaptation based on the overall behavior of the system rather than on pairwise phase and period corrections; thus, they may be more suitable than our model when dealing with situations involving three or more people. The two-alternative forced-choice task adopted for the online experiments may have impaired the resolution of stimulus discrimination. This point is also worth verifying experimentally using confidence rating scales. Despite the above limitations, this study highlights the potential importance of micro-timing in acoustic communications and offers a valuable framework for examining the discriminability of the correlation structures of paired micro-timing fluctuations.

The coupled oscillator model used for stimulus generation is expressed in a form that facilitates comparison with previously established models of discrete phase and period corrections [81–83]. This formulation has the advantage of allowing the easier manipulation of the cross-correlation structure compared to the formulation based on the Kuramoto model [47,49,84]. Additionally, its formulation based on the continuous time evolution of phases enables the simulation of joint rushing, that is, tempo acceleration in an ensemble situation [32,57,85–89], which cannot be replicated by a previous discrete-time linear phase-period correction model [81–83,90], and offers superior properties of ease of expansion to larger ensemble contexts.

It is also noteworthy that the ability to discriminate between stimuli did not correlate with any (sub)scales of the Gold-MSI scale. As the current Gold-MSI lacks a subscale specifically addressing ensemble ability, we directly inquired about the participants’ ensemble experience as a precautionary measure. The results revealed a possible link between the ensemble experience and discrimination ability. Unfortunately, our survey did not clarify how much or what type of ensemble experience enhanced the discrimination ability (participants’ genre backgrounds are summarized in S1 Appendix A Table S1 in the Supporting Information). This limitation arose because we initially hypothesized a correlation between the G-MSI score and *d*’, and to accommodate the online experiment situation, we prioritized minimizing the survey duration. Consequently, the ensemble experience inquiries were restricted to the minimum required for an exploratory analysis. Further research is needed to determine which aspects of the ensemble experience enhance the ability to discriminate between the structures of fluctuation and coordination.

This study quantitatively demonstrated through a systematic listening experiment that discriminability in the timing structures of fluctuations and coordination exists not only in solo classic performances but also in ensembles (at least duos) of isochronous sequences, and that listeners with certain qualities may be able to discriminate between these differences. Although unsolved issues remain, such as the nature of micro-timing and the substantive “humanness” of fluctuation and coordination, the most crucial significance of this study lies in its demonstration that these concepts are not mere illusions perceived by enthusiasts.

## Supporting information

S1 Appendix A Supplementary tables. Tables showing the results of supplementary analyses. (DOCX)

S2 Appendix B Supplementary figures. Scatter plots and correlation matrices for correlations between sensitivity, bias, and G-MSI scores. (DOCX)

S3 File MP3 files of stimuli. (ZIP)

S4 Dataset Data for statistical analyses. (CSV)

## References

[1] Sundberg J, Friberg A, Frydén L. Rules for automated performance of ensemble music. Contemporary Music Review. 1989;3(1):89–109. doi: 10.1080/07494468900640071

[2] Gabrielsson A. The Performance of Music. The Psychology of Music. Elsevier. 1999. p. 501–602. doi: 10.1016/b978-012213564-4/50015-9

[3] Todd N. A Model of Expressive Timing in Tonal Music. Music Perception. 1985;3(1):33–57. doi: 10.2307/40285321

[4] Friberg A, Battel GU. Structural Communication. In: Parncutt R, McPherson G, editors. The Science and Psychology of Music Performance: Creative Strategies for Teaching and Learning. Oxford University Press: Oxford; 2002. p. 199–218.

[5] Friberg A. Matching the rule parameters of PHRASE ARCH to performances of “Träumerei”: a preliminary study. STL-QPSR. 1995;36:063–70.

[6] Rasch RA. Synchronization in performed ensemble music. Acustica. 1979;43:121–31.

[7] Palmer C. On the Assignment of Structure in Music Performance. Music Perception. 1996;14(1):23–56. doi: 10.2307/40285708

[8] Bilmes JA. Timing is of the essence: perceptual and computational techniques for representing, learning, and reproducing expressive timing in percussive rhythm. Thesis (M.S.) in Massachusetts Institute of Technology; 1993. https://dspace.mit.edu/handle/1721.1/62091?show=full

[9] Glen KM. Expressive microtimings and groove in Scottish Gaelic fiddle music. Thesis (M. A.) in University of British Columbia, 2015. 10.14288/1.0166556

[10] Wright M, Berdahl E. Towards machine learning of expressive microtiming in Brazilian drumming. In: Int Conf Math Comput. 2006. https://ccrma.stanford.edu/~eberdahl/Papers/ICMC2006WrightBerdahl.pdf

[11] Iyer V. Embodied Mind, Situated Cognition, and Expressive Microtiming in African-American Music. Music Perception. 2002;19(3):387–414. doi: 10.1525/mp.2002.19.3.387

[12] Sogorski M, Geisel T, Priesemann V. Correlated microtiming deviations in jazz and rock music. PLoS One. 2018;13(1):e0186361. doi: 10.1371/journal.pone.0186361 29364920 PMC5783353

[13] Frühauf J, Kopiez R, Platz F. Music on the timing grid: The influence of microtiming on the perceived groove quality of a simple drum pattern performance. Musicae Scientiae. 2013;17(2):246–60. doi: 10.1177/1029864913486793

[14] Datseris G, Ziereis A, Albrecht T, Hagmayer Y, Priesemann V, Geisel T. Microtiming Deviations and Swing Feel in Jazz. Sci Rep. 2019;9(1):19824. doi: 10.1038/s41598-019-55981-3 31882842 PMC6934603

[15] Jacobsen E, Danielsen A. “Hard” or “Soft”: Shaping Microtiming through Sonic Features in Jazz-Related Groove Performance. Journal of Jazz Studies. 2023.

[16] Davies M, Madison G, Silva P, Gouyon F. The Effect of Microtiming Deviations on the Perception of Groove in Short Rhythms. Music Perception. 2012;30(5):497–510. doi: 10.1525/mp.2013.30.5.497

[17] Danielsen A, Brøvig R, Bøhler KK, Câmara GS, Haugen MR, Jacobsen E. There’s more to timing than time: Investigating musical microrhythm across disciplines and cultures. Music Percept. 2024;41:176–98.

[18] Hennig H, Fleischmann R, Geisel T. Musical rhythms: The science of being slightly off. Physics Today. 2012;65(7):64–5. doi: 10.1063/pt.3.1650

[19] Voss RF, Clarke J. ’’1/f noise’’ in music: Music from 1/f noise. The Journal of the Acoustical Society of America. 1978;63(1):258–63. doi: 10.1121/1.381721

[20] Hsü KJ, Hsü A. Self-similarity of the “1/f noise” called music. Proc Natl Acad Sci U S A. 1991;88:3507–9. 11607178 10.1073/pnas.88.8.3507 PMC51477

[21] Madison G. Variability in isochronous tapping: higher order dependencies as a function of intertap interval. J Exp Psychol Hum Percept Perform. 2001;27(2):411–22. doi: 10.1037//0096-1523.27.2.411 11318056

[22] Wagenmakers E-J, Farrell S, Ratcliff R. Estimation and interpretation of 1/falpha noise in human cognition. Psychon Bull Rev. 2004;11(4):579–615. doi: 10.3758/bf03196615 15581115 PMC1479451

[23] Van Orden GC, Holden JG, Turvey MT. Human cognition and 1/f scaling. J Exp Psychol Gen. 2005;134(1):117–23. doi: 10.1037/0096-3445.134.1.117 15702967

[24] Gilden DL. Cognitive emissions of 1/f noise. Psychol Rev. 2001;108(1):33–56. doi: 10.1037/0033-295x.108.1.33 11212631

[25] VOSS RF, CLARKE J. ‘1/fnoise’ in music and speech. Nature. 1975;258(5533):317–8. doi: 10.1038/258317a0

[26] Gilden DL, Thornton T, Mallon MW. 1/f noise in human cognition. Science. 1995;267(5205):1837–9. doi: 10.1126/science.7892611 7892611

[27] Delignieres D, Ramdani S, Lemoine L, Torre K, Fortes M, Ninot G. Fractal analyses for ‘short’ time series: A re-assessment of classical methods. Journal of Mathematical Psychology. 2006;50(6):525–44. doi: 10.1016/j.jmp.2006.07.004

[28] Hennig H, Fleischmann R, Fredebohm A, Hagmayer Y, Nagler J, Witt A, et al. The nature and perception of fluctuations in human musical rhythms. PLoS One. 2011;6(10):e26457. doi: 10.1371/journal.pone.0026457 22046289 PMC3202537

[29] Hennig H. Synchronization in human musical rhythms and mutually interacting complex systems. Proc Natl Acad Sci U S A. 2014;111(36):12974–9. doi: 10.1073/pnas.1324142111 25114228 PMC4246955

[30] Konvalinka I, Vuust P, Roepstorff A, Frith CD. Follow you, follow me: continuous mutual prediction and adaptation in joint tapping. Q J Exp Psychol (Hove). 2010;63(11):2220–30. doi: 10.1080/17470218.2010.497843 20694920

[31] Okano M, Kurebayashi W, Shinya M, Kudo K. Hybrid dynamics in a paired rhythmic synchronization–continuation task. Physica A: Statistical Mechanics and its Applications. 2019;524:625–38. doi: 10.1016/j.physa.2019.04.102

[32] Okano M, Shinya M, Kudo K. Paired Synchronous Rhythmic Finger Tapping without an External Timing Cue Shows Greater Speed Increases Relative to Those for Solo Tapping. Sci Rep. 2017;7(1). doi: 10.1038/srep43987 PMC5343470 28276461

[33] Marmelat V, Delignières D. Strong anticipation: complexity matching in interpersonal coordination. Exp Brain Res. 2012;222(1–2):137–48. doi: 10.1007/s00221-012-3202-9 22865163

[34] Almurad ZMH, Roume C, Delignières D. Complexity matching in side-by-side walking. Hum Mov Sci. 2017;54:125–36. doi: 10.1016/j.humov.2017.04.008 28460275

[35] Coey CA, Washburn A, Hassebrock J, Richardson MJ. Complexity matching effects in bimanual and interpersonal syncopated finger tapping. Neurosci Lett. 2016;616:204–10. doi: 10.1016/j.neulet.2016.01.066 26840612 PMC4810785

[36] Fine JM, Likens AD, Amazeen EL, Amazeen PG. Emergent complexity matching in interpersonal coordination: Local dynamics and global variability. J Exp Psychol Hum Percept Perform. 2015;41(3):723–37. doi: 10.1037/xhp0000046 25798782

[37] Stephen DG, Stepp N, Dixon JA, Turvey MT. Strong anticipation: Sensitivity to long-range correlations in synchronization behavior. Physica A: Statistical Mechanics and its Applications. 2008;387(21):5271–8. doi: 10.1016/j.physa.2008.05.015

[38] Stephen DG, Dixon JA. Strong anticipation: Multifractal cascade dynamics modulate scaling in synchronization behaviors. Chaos, Solitons & Fractals. 2011;44(1–3):160–8. doi: 10.1016/j.chaos.2011.01.005

[39] Stepp N, Turvey MT. On Strong Anticipation. Cogn Syst Res. 2010;11(2):148–64. doi: 10.1016/j.cogsys.2009.03.003 20191086 PMC2827858

[40] Torre K, Varlet M, Marmelat V. Predicting the biological variability of environmental rhythms: weak or strong anticipation for sensorimotor synchronization?. Brain Cogn. 2013;83(3):342–50. doi: 10.1016/j.bandc.2013.10.002 24212115

[41] Liu HWD. Intentionality of Strong Anticipation in Motor Behaviors. 2010:1930–5.

[42] Friberg A, Sundberg J. Time discrimination in a monotonic, isochronous sequence. The Journal of the Acoustical Society of America. 1995;98(5):2524–31. doi: 10.1121/1.413218

[43] Friberg A, Sundberg J. Perception of just-noticeable time displacement of a tone presented in a metrical sequence at different tempos. The Journal of the Acoustical Society of America. 1993;94(3_Supplement):1859–1859. doi: 10.1121/1.407650

[44] Hirsh IJ. Auditory Perception of Temporal Order. The Journal of the Acoustical Society of America. 1959;31(6):759–67. doi: 10.1121/1.1907782

[45] Pastore RE, Farrington SM. Measuring the difference limen for identification of order of onset for complex auditory stimuli. Percept Psychophys. 1996;58(4):510–26. doi: 10.3758/bf03213087 8934684

[46] Demos AP, Layeghi H, Wanderley MM, Palmer C. Staying Together: A Bidirectional Delay-Coupled Approach to Joint Action. Cogn Sci. 2019;43(8):e12766. doi: 10.1111/cogs.12766 31446664

[47] Heggli OA, Cabral J, Konvalinka I, Vuust P, Kringelbach ML. A Kuramoto model of self-other integration across interpersonal synchronization strategies. PLoS Comput Biol. 2019;15(10):e1007422. doi: 10.1371/journal.pcbi.1007422 31618261 PMC6816575

[48] Demos AP, Palmer C. Social and nonlinear dynamics unite: musical group synchrony. Trends Cogn Sci. 2023;27(11):1008–18. doi: 10.1016/j.tics.2023.05.005 37277276

[49] Dotov D, Delasanta L, Cameron DJ, Large EW, Trainor L. Collective dynamics support group drumming, reduce variability, and stabilize tempo drift. Elife. 2022;11:e74816. doi: 10.7554/eLife.74816 36317963 PMC9678363

[50] Goebl W, Palmer C. Synchronization of Timing and Motion Among Performing Musicians. Music Perception. 2009;26(5):427–38. doi: 10.1525/mp.2009.26.5.427

[51] Bishop L, Cancino-Chacón C, Goebl W. Moving to communicate, moving to interact: Patterns of body motion in musical duo performance. Music Percept. 2019;37:1–25.

[52] Palmer C, Spidle F, Koopmans E, Schubert P. Ears, heads, and eyes: When singers synchronise. Q J Exp Psychol (Hove). 2019;72(9):2272–87. doi: 10.1177/1747021819833968 30744490

[53] Loehr JD, Palmer C. Cognitive and biomechanical influences in pianists’ finger tapping. Exp Brain Res. 2007;178(4):518–28. doi: 10.1007/s00221-006-0760-8 17093990

[54] Volpe G, D’Ausilio A, Badino L, Camurri A, Fadiga L. Measuring social interaction in music ensembles. Philos Trans R Soc Lond B Biol Sci. 2016;371(1693):20150377. doi: 10.1098/rstb.2015.0377 27069054 PMC4843615

[55] Okano M, Kurebayashi W, Shinya M, Kudo K. A coupled oscillator model for acceleration of a paired tapping through mutual timing adjustment for synchronization. In: Studies in Perception and Action XIV: Nineteenth International Conference on Perception and Action. 2017. 21–4.

[56] Roume C, Almurad ZMH, Scotti M, Ezzina S, Blain H, Delignières D. Windowed detrended cross-correlation analysis of synchronization processes. Physica A: Statistical Mechanics and its Applications. 2018;503:1131–50. doi: 10.1016/j.physa.2018.08.074

[57] Konvalinka I, Vuust P, Roepstorff A, Frith C. A coupled oscillator model of interactive tapping. In: Proceedings of the 7th Triennial Conference of European Society for the Cognitive Sciences of Music (ESCOM 2009). 2009. 242–5. https://jyx.jyu.fi/dspace/handle/123456789/20884

[58] Eerola T, Toiviainen P. MIDI toolbox: MATLAB tools for music research. Department of Music, University of Jyväskylä; 2004.

[59] Sadakata M, Yamaguchi Y, Ohsawa C, Matsubara M, Terasawa H, von Schnehen A, et al. The Japanese translation of the Gold-MSI: Adaptation and validation of the self-report questionnaire of musical sophistication. Musicae Scientiae. 2022;27(3):798–810. doi: 10.1177/10298649221110089

[60] Müllensiefen D, Gingras B, Musil J, Stewart L. The musicality of non-musicians: an index for assessing musical sophistication in the general population. PLoS One. 2014;9(2):e89642. doi: 10.1371/journal.pone.0089642 24586929 PMC3935919

[61] Miyazaki K, Hiraga Y, Adachi M, Nakajima Y, Tsuzaki M. The Beat Alignment Test (BAT): Surveying beat processing abilities in the general population. In: Proceedings of the 10th International Conference on Music Perception and Cognition. 2008. https://www.researchgate.net/profile/John-Iversen-2/publication/228483453_The_Beat_Alignment_Test_BAT_Surveying_beat_processing_abilities_in_the_general_population/links/00b7d5233b33d2bd39000000/The-Beat-Alignment-Test-BAT-Surveying-beat-processing-abilities-in-the-general-population.pdf

[62] Harrison PMC, Müllensiefen D. Development and Validation of the Computerised Adaptive Beat Alignment Test (CA-BAT). Sci Rep. 2018;8(1):12395. doi: 10.1038/s41598-018-30318-8 30120265 PMC6097996

[63] Fujii S, Schlaug G. Corrigendum: The Harvard Beat Assessment Test (H-BAT): a battery for assessing beat perception and production and their dissociation. Front Hum Neurosci. 2014;8:870. doi: 10.3389/fnhum.2014.00870 25406802 PMC4219433

[64] Henninger F, Shevchenko Y, Mertens UK, Kieslich PJ, Hilbig BE. lab.js: A free, open, online study builder. Behav Res Methods. 2022;54(2):556–73. doi: 10.3758/s13428-019-01283-5 34322854 PMC9046347

[65] Woods KJP, Siegel MH, Traer J, McDermott JH. Headphone screening to facilitate web-based auditory experiments. Atten Percept Psychophys. 2017;79(7):2064–72. doi: 10.3758/s13414-017-1361-2 28695541 PMC5693749

[66] Anduiza E, Galais C. Answering Without Reading: IMCs and Strong Satisficing in Online Surveys. Int J Public Opin Res. 2016;:edw007. doi: 10.1093/ijpor/edw007

[67] Roberts C, Gilbert E, Allum N, Eisner L. Research Synthesis. Public Opinion Quarterly. 2019;83(3):598–626. doi: 10.1093/poq/nfz035

[68] Gescheider G. Psychophysics: The Fundamentals. 1997. doi: 10.4324/9780203774458/psychophysics-george-gescheider

[69] Schielzeth H, Dingemanse NJ, Nakagawa S, Westneat DF, Allegue H, Teplitsky C, et al. Robustness of linear mixed‐effects models to violations of distributional assumptions. Methods Ecol Evol. 2020;11(9):1141–52. doi: 10.1111/2041-210x.13434

[70] Jacqmin-Gadda H, Sibillot S, Proust C, Molina J-M, Thiébaut R. Robustness of the linear mixed model to misspecified error distribution. Computational Statistics & Data Analysis. 2007;51(10):5142–54. doi: 10.1016/j.csda.2006.05.021

[71] Bates D, Mächler M, Bolker B, Walker S. Fitting Linear Mixed-Effects Models Usinglme4. J Stat Soft. 2015;67(1). doi: 10.18637/jss.v067.i01

[72] Kuznetsova A, Brockhoff PB, Christensen RHB. lmerTest Package: Tests in Linear Mixed Effects Models. J Stat Soft. 2017;82(13). doi: 10.18637/jss.v082.i13

[73] Stoffel MA, Nakagawa S, Schielzeth H. partR2: partitioning R2 in generalized linear mixed models. PeerJ. 2021;9:e11414. doi: 10.7717/peerj.11414 34113487 PMC8162244

[74] Nakagawa S, Schielzeth H. A general and simple method for obtaining R2 from generalized linear mixed‐effects models. Methods Ecol Evol. 2012;4(2):133–42. doi: 10.1111/j.2041-210x.2012.00261.x

[75] Ben-Shachar M, Lüdecke D, Makowski D. effectsize: Estimation of Effect Size Indices and Standardized Parameters. JOSS. 2020;5(56):2815. doi: 10.21105/joss.02815

[76] Pustejovsky JE, Tipton E. Small-Sample Methods for Cluster-Robust Variance Estimation and Hypothesis Testing in Fixed Effects Models. Journal of Business & Economic Statistics. 2017;36(4):672–83. doi: 10.1080/07350015.2016.1247004

[77] Repp BH. Sensorimotor synchronization and perception of timing: effects of music training and task experience. Hum Mov Sci. 2010;29(2):200–13. doi: 10.1016/j.humov.2009.08.002 20074825

[78] Hove MJ, Marie C, Bruce IC, Trainor LJ. Superior time perception for lower musical pitch explains why bass-ranged instruments lay down musical rhythms. Proc Natl Acad Sci U S A. 2014;111(28):10383–8. doi: 10.1073/pnas.1402039111 24982142 PMC4104866

[79] Gordon JW. The perceptual attack time of musical tones. J Acoust Soc Am. 1987;82(1):88–105. doi: 10.1121/1.395441 3624645

[80] Van Kerrebroeck B, Wanderley MM, Demos AP, Palmer C. Virtual Partners Improve Synchronization in Human-Machine Trios. Cogn Sci. 2025;49(2):e70040. doi: 10.1111/cogs.70040 39898831

[81] Mates J. A model of synchronization of motor acts to a stimulus sequence. I. Timing and error corrections. Biol Cybern. 1994;70(5):463–73. doi: 10.1007/BF00203239 8186306

[82] Vorberg D, Schulze H-H. Linear Phase-Correction in Synchronization: Predictions, Parameter Estimation, and Simulations. Journal of Mathematical Psychology. 2002;46(1):56–87. doi: 10.1006/jmps.2001.1375

[83] Repp BH, Keller PE. Sensorimotor synchronization with adaptively timed sequences. Hum Mov Sci. 2008;27(3):423–56. doi: 10.1016/j.humov.2008.02.016 18405989

[84] Kuramoto Y. Self-entrainment of a population of coupled non-linear oscillators. Lecture Notes in Physics. Springer-Verlag. p. 420–2. doi: 10.1007/bfb0013365

[85] Thomson M, Murphy K, Lukeman R. Groups clapping in unison undergo size-dependent error-induced frequency increase. Sci Rep. 2018;8(1):808. doi: 10.1038/s41598-017-18539-9 29339736 PMC5770382

[86] van de Rijt A. All-sense-all networks are suboptimal for sensorimotor synchronization. PLoS One. 2018;13(8):e0202056. doi: 10.1371/journal.pone.0202056 30157192 PMC6114297

[87] Wolf T, Vesper C, Sebanz N, Keller PE, Knoblich G. Combining Phase Advancement and Period Correction Explains Rushing during Joint Rhythmic Activities. Sci Rep. 2019;9(1):9350. doi: 10.1038/s41598-019-45601-5 31249346 PMC6597726

[88] Wolf T, Knoblich G. Joint rushing alters internal timekeeping in non-musicians and musicians. Sci Rep. 2022;12(1):1190. doi: 10.1038/s41598-022-05298-5 35075243 PMC8786930

[89] Koike Y, Ogata T, Nozawa T, Miyake Y. Effect of time delay on performance and timing control in dyadic rhythm coordination using finger tapping. Sci Rep. 2024;14(1):17382. doi: 10.1038/s41598-024-68326-6 39075177 PMC11286935

[90] Wing AM, Endo S, Bradbury A, Vorberg D. Optimal feedback correction in string quartet synchronization. J R Soc Interface. 2014;11(93):20131125. doi: 10.1098/rsif.2013.1125 24478285 PMC3928944

