# Supplementary material for: Coupled-oscillator-humanizer revealed possible ensemble players’ ability to discriminate cross-correlation structures in auditory sequences of paired drum tapping
Source: PLoS One. 2025 Nov 20;20(11):e0336778. doi: 10.1371/journal.pone.0336778 (PMC12633884; doi:10.1371/journal.pone.0336778)
Supplement: S1 Appendix A — (DOCX) [file pone.0336778.s001.docx]

**Supplementary Materials**

**Appendix A – supplementary tables**

**Cross-correlation structures of stimuli**

The setting of model parameter *b* was determined based on the windowed detrended cross-correlation coefficients (WDCC) at lags of -1, 0, and 1, the core cross-correlation structure of paired synchronous tapping. The cross-correlation structure at *b* = 1.5 was confirmed to be similar to the most prominent example reported in Supplementary Table S4 of Okano et al. (2019), while the other two levels (*b* = 1.0 and 0.5) were set to represent moderate and weak period corrections. Table S1 and S2 shows the WDCC coefficients for HUM and RAN, respectively.

Table S1. Windowed detrended cross-correlation coefficients for each lag and *b* (HUM stimuli).

| StimuliNo. |  | *b* = 1.5 | | |  | *b* = 1.0 | | |  | *b* = 0.5 | | |
| --- | --- | --- | --- | --- | --- | --- | --- | --- | --- | --- | --- | --- |
|  |  | lag -1 | lag 0 | lag 1 |  | lag -1 | lag 0 | lag 1 |  | lag -1 | lag 0 | lag 1 |
| 1 |  | 0.46 | -0.09 | 0.25 |  | 0.16 | -0.25 | 0.15 |  | 0.19 | -0.42 | 0.32 |
| 2 |  | 0.39 | -0.61 | 0.45 |  | 0.12 | -0.20 | 0.05 |  | -0.02 | -0.07 | 0.08 |
| 3 |  | 0.54 | -0.34 | 0.55 |  | 0.11 | -0.02 | 0.20 |  | 0.28 | -0.50 | 0.15 |
| 4 |  | 0.03 | -0.60 | 0.52 |  | 0.22 | -0.20 | 0.38 |  | 0.12 | 0.14 | -0.04 |
| 5 |  | 0.28 | -0.47 | 0.34 |  | -0.05 | -0.06 | 0.23 |  | 0.07 | -0.11 | -0.28 |
| 6 |  | 0.17 | -0.38 | 0.31 |  | -0.07 | -0.22 | 0.35 |  | -0.08 | -0.24 | 0.16 |
| 7 |  | 0.45 | -0.63 | 0.53 |  | 0.27 | 0.00 | 0.02 |  | 0.00 | -0.06 | -0.08 |
| 8 |  | 0.36 | -0.44 | 0.32 |  | 0.28 | -0.21 | 0.05 |  | 0.14 | -0.06 | 0.28 |
| 9 |  | 0.06 | -0.33 | 0.23 |  | 0.01 | -0.49 | 0.01 |  | -0.05 | -0.13 | 0.24 |
| 10 |  | -0.08 | -0.32 | 0.58 |  | -0.18 | -0.10 | 0.12 |  | 0.10 | -0.38 | 0.01 |
| 11 |  | 0.30 | -0.17 | 0.45 |  | 0.44 | -0.02 | 0.15 |  | 0.03 | -0.19 | 0.12 |
| 12 |  | 0.23 | -0.11 | 0.18 |  | 0.21 | -0.05 | 0.23 |  | -0.31 | -0.12 | -0.05 |
| 13 |  | 0.46 | -0.60 | 0.63 |  | 0.25 | -0.40 | 0.23 |  | 0.30 | -0.11 | -0.19 |
| 14 |  | 0.50 | -0.56 | 0.31 |  | 0.18 | -0.22 | 0.45 |  | -0.15 | -0.24 | 0.14 |
| 15 |  | 0.78 | -0.62 | 0.42 |  | 0.32 | 0.00 | 0.32 |  | 0.13 | -0.10 | 0.12 |
| 16 |  | 0.18 | -0.39 | 0.45 |  | 0.19 | -0.24 | 0.19 |  | 0.11 | -0.03 | -0.07 |
| 17 |  | 0.34 | -0.48 | 0.21 |  | 0.26 | -0.15 | 0.02 |  | 0.56 | -0.18 | -0.20 |
| 18 |  | 0.39 | -0.61 | 0.53 |  | -0.02 | -0.08 | 0.27 |  | -0.06 | -0.15 | 0.16 |
| 19 |  | 0.27 | -0.32 | 0.25 |  | -0.16 | 0.04 | -0.03 |  | 0.12 | -0.15 | 0.17 |
| 20 |  | 0.14 | -0.42 | 0.43 |  | 0.08 | 0.15 | 0.09 |  | -0.04 | -0.41 | -0.05 |
| Mean |  | 0.31 | -0.43 | 0.40 |  | 0.13 | -0.14 | 0.17 |  | 0.07 | -0.18 | 0.05 |
| Std |  | 0.20 | 0.17 | 0.13 |  | 0.16 | 0.15 | 0.13 |  | 0.18 | 0.15 | 0.16 |

Table S2. Windowed detrended cross-correlation coefficients for each lag and *b* (RAN stimuli).

| StimuliNo. |  | *b* = 1.5 | | |  | *b* = 1.0 | | |  | *b* = 0.5 | | |
| --- | --- | --- | --- | --- | --- | --- | --- | --- | --- | --- | --- | --- |
|  |  | lag -1 | lag 0 | lag 1 |  | lag -1 | lag 0 | lag 1 |  | lag -1 | lag 0 | lag 1 |
| 1 |  | 0.11 | 0.20 | 0.24 |  | -0.10 | 0.17 | 0.04 |  | 0.36 | -0.25 | 0.33 |
| 2 |  | 0.17 | -0.26 | 0.31 |  | 0.11 | -0.09 | 0.38 |  | 0.22 | -0.01 | -0.07 |
| 3 |  | 0.17 | 0.14 | 0.18 |  | 0.34 | 0.11 | -0.12 |  | 0.21 | -0.33 | -0.01 |
| 4 |  | -0.24 | -0.24 | 0.15 |  | 0.08 | 0.09 | 0.30 |  | 0.12 | 0.13 | -0.07 |
| 5 |  | 0.27 | -0.27 | 0.44 |  | 0.14 | -0.26 | 0.19 |  | -0.01 | 0.22 | -0.06 |
| 6 |  | 0.29 | -0.11 | -0.17 |  | 0.25 | -0.08 | 0.31 |  | -0.13 | -0.19 | 0.27 |
| 7 |  | 0.03 | 0.08 | 0.23 |  | 0.10 | 0.01 | 0.41 |  | 0.11 | 0.13 | -0.17 |
| 8 |  | 0.04 | -0.30 | 0.33 |  | 0.24 | -0.18 | -0.05 |  | 0.12 | -0.10 | 0.22 |
| 9 |  | 0.19 | -0.19 | -0.02 |  | 0.00 | 0.08 | -0.08 |  | 0.28 | 0.00 | 0.34 |
| 10 |  | 0.09 | 0.11 | 0.17 |  | 0.21 | -0.16 | 0.38 |  | 0.29 | -0.34 | -0.06 |
| 11 |  | 0.03 | 0.31 | 0.02 |  | 0.36 | 0.04 | -0.11 |  | 0.22 | -0.14 | 0.26 |
| 12 |  | -0.06 | -0.11 | 0.04 |  | -0.12 | -0.02 | 0.28 |  | 0.19 | 0.06 | -0.05 |
| 13 |  | 0.09 | -0.26 | 0.07 |  | -0.28 | 0.02 | 0.27 |  | 0.19 | -0.07 | -0.20 |
| 14 |  | -0.06 | -0.01 | 0.31 |  | 0.34 | -0.15 | 0.20 |  | 0.22 | 0.02 | -0.11 |
| 15 |  | 0.40 | 0.22 | -0.17 |  | 0.41 | 0.19 | 0.26 |  | 0.07 | -0.14 | 0.34 |
| 16 |  | 0.04 | -0.02 | 0.01 |  | 0.10 | 0.00 | 0.10 |  | 0.15 | -0.05 | 0.26 |
| 17 |  | 0.18 | -0.13 | 0.30 |  | 0.08 | -0.17 | 0.33 |  | 0.35 | -0.10 | 0.12 |
| 18 |  | 0.07 | -0.32 | 0.09 |  | -0.03 | 0.24 | 0.01 |  | -0.01 | -0.19 | 0.19 |
| 19 |  | 0.29 | -0.42 | 0.13 |  | -0.03 | -0.16 | 0.04 |  | 0.14 | -0.19 | 0.13 |
| 20 |  | -0.15 | 0.00 | 0.05 |  | 0.41 | -0.24 | 0.23 |  | -0.22 | -0.31 | 0.27 |
| mean |  | 0.10 | -0.08 | 0.13 |  | 0.13 | -0.03 | 0.17 |  | 0.14 | -0.09 | 0.10 |
| std |  | 0.15 | 0.20 | 0.16 |  | 0.19 | 0.14 | 0.17 |  | 0.14 | 0.15 | 0.18 |

# **Data screening using “human-like” response to ISO**

In the main experiment, ISO stimuli, i.e., easily discriminable isochronous auditory rhythm stimuli, were presented six times per participant to identify satisficing and inattentive participants. Table S3 shows the frequency of “human-like” responses to ISO and the participant counts for each model parameter *b* and ensemble experience. Over 90% of participants judged ISO as “human-like” once or fewer times. Consequently, participants who judged ISO as “human-like” twice or more were excluded from the subsequent statistical analyses as these participants were considered inattentive.

Table S3. Frequency and counts of participants who answered “human” to ISO.

| *b* | | 1.5 | | 1.0 | | 0.5 | |
| --- | --- | --- | --- | --- | --- | --- | --- |
| Ensemble experience | | Yes | No | Yes | No | Yes | No |
| Frequency | 0 | 18 | 65 | 24 | 65 | 22 | 57 |
|  | 1 | 3 | 14 | 0 | 11 | 0 | 8 |
|  | 2 | 0 | 2 | 0 | 0 | 2 | 1 |
|  | 3 | 1 | 0 | 0 | 3 | 0 | 1 |
|  | 4 | 0 | 1 | 0 | 1 | 0 | 1 |
|  | 5 | 0 | 0 | 0 | 2 | 0 | 2 |
|  | 6 | 0 | 1 | 0 | 0 | 0 | 0 |

**Music genres of participants with ensemble experience**

In the survey at the end of the experiment, participants were asked whether they regularly participated in an ensemble (yes/no selection and, if yes, genre selection). Table S4 presents the genre options alongside the number of participants.

Table S4. Music genres and corresponding number of participants with ensemble experience.

| *b* | 1.5 | 1.0 | 0.5 |
| --- | --- | --- | --- |
| A cappella | 1 | 0 | 0 |
| Brass band | 7 | 7 | 6 |
| Choir | 3 | 2 | 5 |
| Electric music | 1 | 0 | 0 |
| Jazz band | 0 | 1 | 2 |
| Rock band | 1 | 11 | 6 |
| Others | 8 | 3 | 3 |
| Total | 21 | 24 | 22 |

**Robust estimates and effect size**

In linear mixed model (LMM) analyses, the assumption of normality of residuals was violated in the analyses of GMSI and C (response bias), and missing random effects occurred in all analyses. Although fixed effect estimates have been demonstrated to be robust to these violations, the results of tests using robust standard errors are summarized in Tables S5-S9 (corresponding to Tables 3-7 in the main text), for reference.

Table S5. Robust estimates and effect sizes for fixed effects on G-MSI score.

| Term | Estimate | Robust SE | *df* | *t* | *p* | Stdardized coefficient | [95% CI] | Inclusive R^2^ | [95% CI] |
| --- | --- | --- | --- | --- | --- | --- | --- | --- | --- |
| Intercept | 136.63 | 2.68 | 72.65 | 51.08 | < .001^*^ | 0.30 | [0.157, 0.449] | - | - |
| *b* (0.5) | 1.33 | 0.50 | 20.46 | 2.65 | .015^*^ | 0.04 | [0.002, 0.074] | .001 | [0, 0.003] |
| *b* (1.0) | -0.77 | 0.55 | 17.81 | -1.40 | .178 | -0.02 | [-0.058, 0.014] | < .000 | [0, 0.001] |
| Ensemble (no) | -16.22 | 2.68 | 72.65 | -6.06 | < .001^*^ | -0.46 | [-0.61, -0.319] | .175 | [0.084, 0.256] |
| *b* (0.5) × Ensemble (no) | -0.32 | 0.50 | 20.46 | -0.64 | .526 | -0.01 | [-0.045, 0.027] | < .001 | [0, 0.001] |
| *b* (1.0) ×Ensemble (no) | 0.08 | 0.55 | 17.81 | 0.14 | .887 | 0.00 | [-0.034, 0.039] | < .001 | [0, 0.001] |

^*^: *p* < .05

Table S6. Robust estimates and effect sizes for fixed effects on “human-like” response rate.

| Term | Estimate | Robust SE | *df* | *t* | *p* | Stdardized coefficient | [95% CI] | Inclusive R^2^ | [95% CI] |
| --- | --- | --- | --- | --- | --- | --- | --- | --- | --- |
| Intercept | 52.26 | 0.93 | 69.15 | 55.99 | < .001^*^ | -0.02 | [-0.147, 0.115] | - | - |
| Stimuli (HUM) | 1.37 | 0.51 | 55.83 | 2.69 | .009^*^ | 0.09 | [0.015, 0.173] | .003 | [0, 0.015] |
| *b* (0.5) | -0.30 | 0.90 | 37.03 | -0.33 | .741 | -0.02 | [-0.151, 0.11] | .001 | [0, 0.011] |
| *b* (1.0) | -0.82 | 0.74 | 37.64 | -1.11 | .274 | -0.06 | [-0.185, 0.073] | .001 | [0, 0.012] |
| Ensemble (no) | 1.33 | 0.93 | 69.15 | 1.43 | .158 | 0.09 | [-0.039, 0.222] | .006 | [0, 0.034] |
| Stimuli (HUM) × *b* (0.5) | -1.23 | 0.76 | 49.13 | -1.61 | .114 | -0.08 | [-0.197, 0.029] | .005 | [0, 0.019] |
| Stimuli (HUM) × *b* (1.0) | 0.93 | 0.86 | 53.31 | 1.07 | .289 | 0.06 | [-0.046, 0.173] | < .001 | [0, 0.006] |
| Stimuli (HUM)  × Ensemble (no) | -1.14 | 0.51 | 55.83 | -2.24 | .029^*^ | -0.08 | [-0.157, 0.001] | < .001 | [0, 0.007] |
| *b* (0.5) × Ensemble (no) | -0.18 | 0.90 | 37.03 | -0.20 | .843 | -0.01 | [-0.143, 0.118] | < .001 | [0, 0.007] |
| *b* (1.0) × Ensemble (no) | 0.79 | 0.74 | 37.64 | 1.08 | .288 | 0.05 | [-0.075, 0.183] | < .001 | [0, 0.007] |
| Stimuli (HUM) × *b* (0.5)  × Ensemble (no) | -0.91 | 0.76 | 49.13 | -1.19 | .238 | -0.06 | [-0.175, 0.05] | .006 | [0, 0.020] |
| Stimuli (HUM) × *b* (1.0)  × Ensemble (no) | -0.26 | 0.86 | 53.31 | -0.30 | .764 | -0.02 | [-0.128, 0.092] | .001 | [0, 0.01] |

^*^: *p* < .05

Table S7. Robust estimates and effect sizes for fixed effects on *d*’ (sensitivity).

| Term | Estimate | Robust SE | *df* | *t* | *p* | Stdardized coefficient | [95% CI] | Inclusive R^2^ | [95% CI] |
| --- | --- | --- | --- | --- | --- | --- | --- | --- | --- |
| Intercept | 0.01 | 0.00 | 55.83 | 2.66 | .010^*^ | 0.06 | [-0.081, 0.192] | - | - |
| *b* (0.5) | -0.01 | 0.01 | 49.13 | -1.58 | .120 | -0.14 | [-0.331, 0.058] | .013 | [0, 0.054] |
| *b* (1.0) | 0.01 | 0.01 | 53.31 | 0.99 | .326 | 0.10 | [-0.091, 0.288] | < .000 | [0, 0.016] |
| Ensemble (no) | -0.01 | 0.00 | 55.83 | -2.19 | .033^*^ | -0.13 | [-0.264, 0.009] | .011 | [0, 0.048] |
| *b* (0.5) × Ensemble (no) | -0.01 | 0.01 | 49.13 | -1.25 | .216 | -0.11 | [-0.303, 0.087] | .016 | [0, 0.057] |
| *b* (1.0) × Ensemble (no) | 0.00 | 0.01 | 53.31 | -0.24 | .814 | -0.02 | [-0.213, 0.166] | .003 | [0, 0.027] |

^*^: *p* < .05

Table S8. Robust estimates and effect sizes for fixed effects on *C* (response bias).

| Term | Estimate | Robust SE | *df* | *t* | *p* | Stdardized coefficient | [95% CI] | Inclusive R^2^ | [95% CI] |
| --- | --- | --- | --- | --- | --- | --- | --- | --- | --- |
| Intercept | 0.68 | 0.00 | 70.21 | 208.34 | < .001^*^ | 0.02 | [-0.147, 0.183] | - | - |
| *b* (0.5) | 0.00 | 0.00 | 34.23 | 0.29 | .774 | 0.02 | [-0.129, 0.173] | .002 | [0, 0.015] |
| *b* (1.0) | 0.00 | 0.00 | 34.07 | 1.21 | .235 | 0.08 | [-0.075, 0.225] | .002 | [0, 0.017] |
| Ensemble (no) | 0.00 | 0.00 | 70.21 | -1.52 | .133 | -0.12 | [-0.285, 0.045] | .011 | [0, 0.055] |
| *b* (0.5) × Ensemble (no) | 0.00 | 0.00 | 34.23 | 0.23 | .822 | 0.02 | [-0.133, 0.168] | < .001 | [0, 0.010] |
| *b* (1.0) × Ensemble (no) | 0.00 | 0.00 | 34.07 | -1.02 | .315 | -0.06 | [-0.213, 0.086] | < .001 | [0, 0.010] |

^*^: *p* < .05

Table S9. Robust estimates and effect sizes for fixed effects on accurate response rate in practice trials.

| Term | Estimate | Robust SE | *df* | *t* | *p* | Stdardized coefficient | [95% CI] | Inclusive R^2^ | [95% CI] |
| --- | --- | --- | --- | --- | --- | --- | --- | --- | --- |
| Intercept | 0.66 | 0.01 | 55.83 | 82.56 | < .001^*^ | 0.04 | [-0.102, 0.174] | - | - |
| *b* (0.5) | 0.00 | 0.01 | 49.13 | -0.03 | .980 | 0.00 | [-0.199, 0.195] | .001 | [0, 0.0208] |
| *b* (1.0) | -0.02 | 0.01 | 53.31 | -1.49 | .141 | -0.13 | [-0.32, 0.064] | .003 | [0, 0.028] |
| Ensemble (no) | -0.01 | 0.01 | 55.83 | -1.14 | .260 | -0.06 | [-0.203, 0.073] | .002 | [0, 0.027] |
| *b* (0.5) × Ensemble (no) | 0.00 | 0.01 | 49.13 | -0.17 | .864 | -0.01 | [-0.212, 0.182] | < .000 | [0, 0.020] |
| *b* (1.0) × Ensemble (no) | 0.02 | 0.01 | 53.31 | 1.44 | .155 | 0.12 | [-0.068, 0.316] | .001 | [0, 0.025] |

^*^: *p* < .05
