# Supplementary material for: Coupled-oscillator-humanizer revealed possible ensemble players’ ability to discriminate cross-correlation structures in auditory sequences of paired drum tapping
Source: PLoS One. 2025 Nov 20;20(11):e0336778. doi: 10.1371/journal.pone.0336778 (PMC12633884; doi:10.1371/journal.pone.0336778)
Supplement: S2 Appendix B — (DOCX) [file pone.0336778.s002.docx]

**Supplementary Materials**

**Appendix B – correlation among sensitivity, bias, and G-MSI scores.**

Correlations among accuracy rate in practice trials, *d*’, *C*, and G-MSI scores were examined using Pearson’s correlation coefficient. The correlations were weak and not consistent among different *b* (see Figures S1-S6).

Variables:

Acc. prac. … Accuracy rate in practice trials.

d’ … Sensitivity.

C … Bias.

Total … Total G-MSI score.

Act … Active engagement subscale in G-MSI.

Percep … Perceptual abilities subscale in G-MSI.

Train … Musical training subscale in G-MSI.

Sing … Singing abilities subscale in G-MSI.

Emo … Emotions subscale in G-MSI.


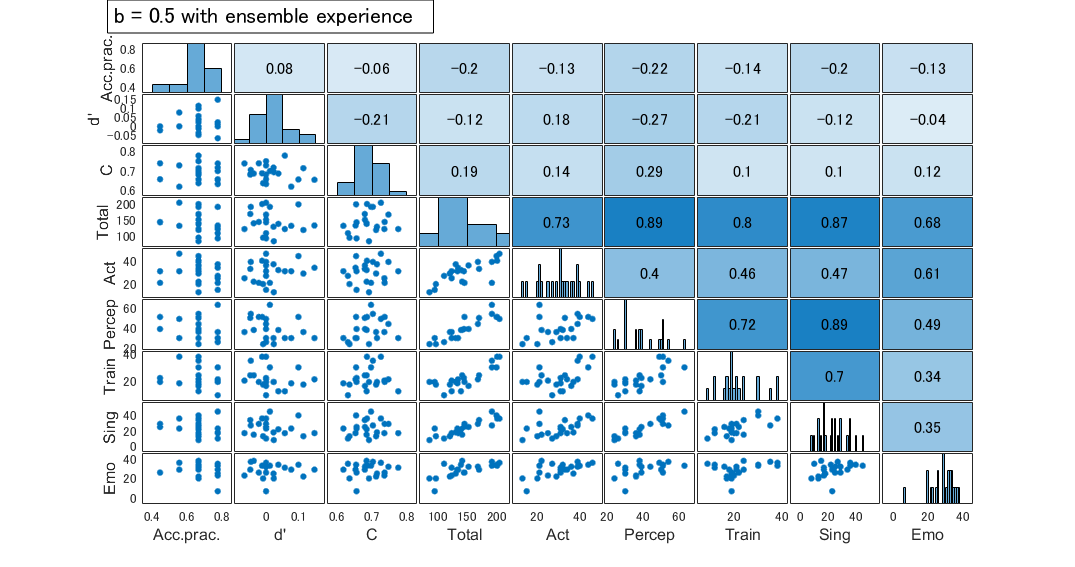


Figure S 1 Scatter plot matrix for parameter *b* = 0.5 (participants with ensemble experience). Pearson correlations are reported in the corresponding upper-triangle panels.


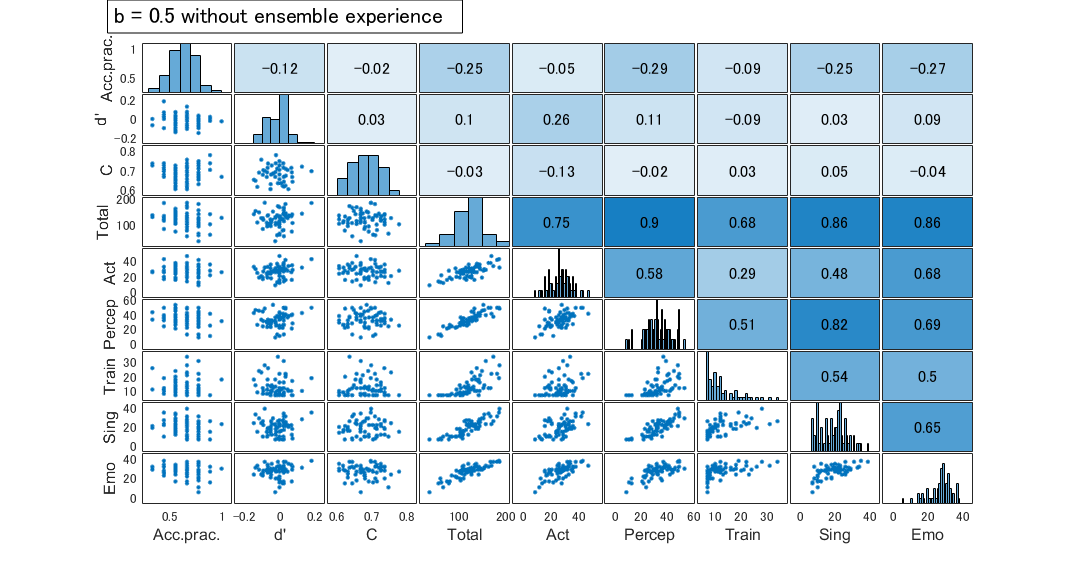


Figure S 2 Scatter plot matrix for parameter *b* = 0.5 (participants without ensemble experience). Pearson correlations are reported in the corresponding upper-triangle panels.


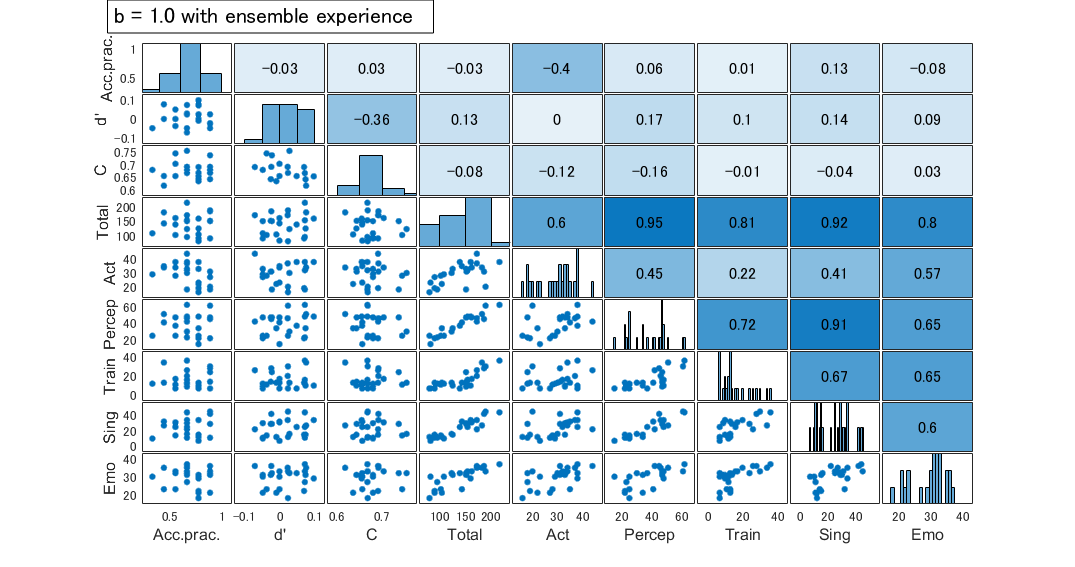


Figure S 3 Scatter plot matrix for parameter *b* = 1.0 (participants with ensemble experience). Pearson correlations are reported in the corresponding upper-triangle panels.


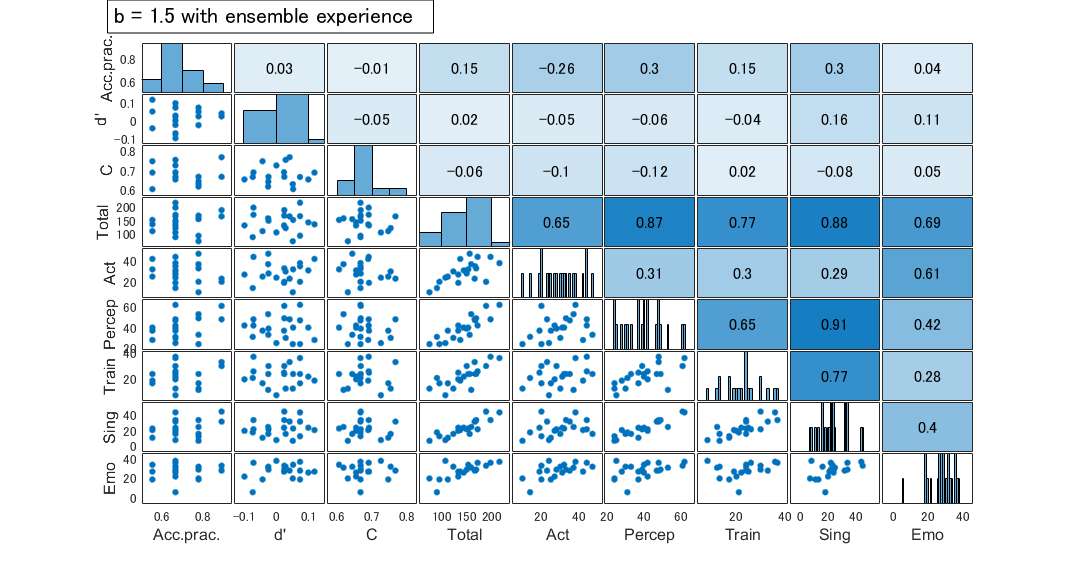

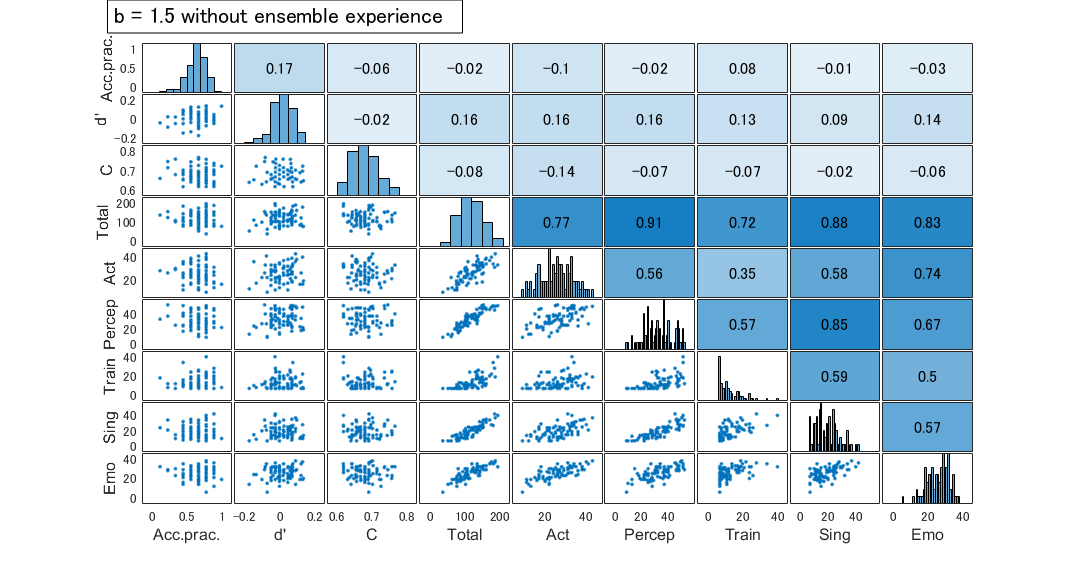


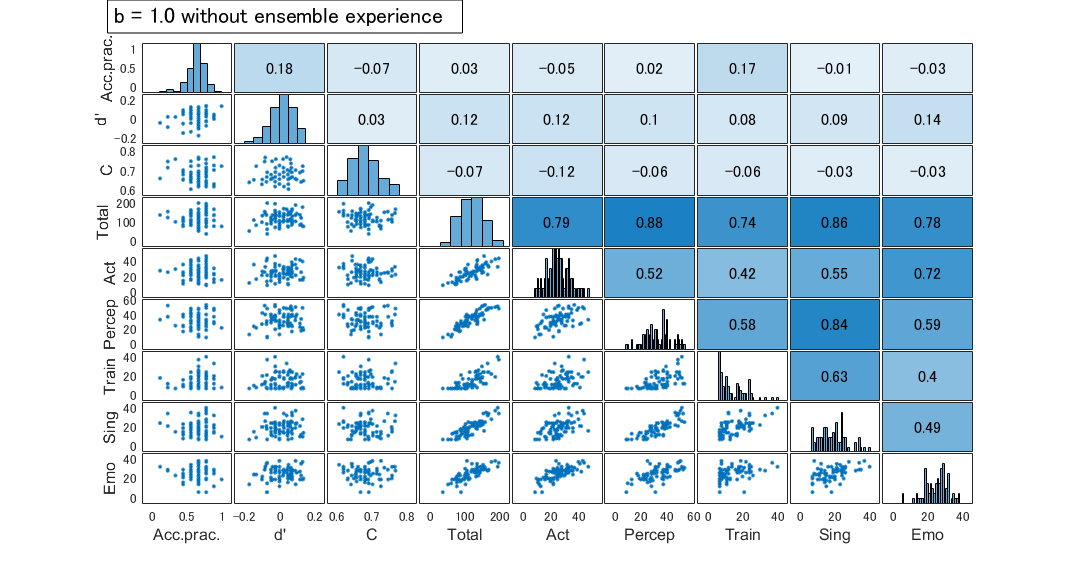


Figure S 4 Scatter plot matrix for parameter *b* = 1.0 (participants without ensemble experience). Pearson correlations are reported in the corresponding upper-triangle panels.

Figure S 5 Scatter plot matrix for parameter *b* = 1.5 (participants with ensemble experience). Pearson correlations are reported in the corresponding upper-triangle panels.

Figure S 6 Scatter plot matrix for parameter *b* = 1.5 (participants without ensemble experience). Pearson correlations are reported in the corresponding upper-triangle panels.
